# Interventional impact of liposomal iron on iron-deficient children developmental outcome: randomized, double-blind, placebo-controlled trial

**DOI:** 10.1038/s41390-025-04204-9

**Published:** 2025-06-20

**Authors:** Wael A. Bahbah, Zein A. Omar, Ali M. El-Shafie, Kerollos S. Mahrous, Heba M. S. El Zefzaf

**Affiliations:** Department of Pediatrics, Faculty of Medicine, Menoufia University, Shebin El-Kom, Egypt

## Abstract

**Background:**

Iron deficiency (ID) in children, especially those under five years, has a well-recognized negative impact on development and growth. However, the potential for reversibility with iron supplementation remains debatable. Therefore, we aimed in this double-blind placebo-controlled clinical trial to investigate the effect of the novel iron preparation, Liposomal Iron (LI), on the development and growth of iron-deficient children.

**Methods:**

For iron deficiency anemia (IDA) and non-anemic iron deficiency (NAID), 433 children aged 6 to 59 months underwent screening. The NAID group was divided into two groups: one group received a placebo, while the interventional group received liposomal iron for four months, as in the IDA group. Then, compared to baseline, efficacy, development, and growth were reevaluated.

**Results:**

The NAID interventional group showed significant improvement in developmental total scores compared to the NAID placebo group with a mean difference of +29.57 versus +0.96 (*P* < 0.001). Furthermore, the final score was notably superior in the NAID interventional group 181.5, compared to the IDA group, 175.0 (*P* < 0.001).

**Conclusions:**

Liposomal iron showed, in addition to good efficacy and tolerability, a favorable impact on the development and growth of iron-deficient children. However, the best results were obtained from early management prior to the onset of anemia (NAID).

**Impact:**

- NAID, though common, is still underappreciated, and evidence about the benefits of giving iron to children before IDA progression is limited.
- The Ages and Stages Questionnaires is a reliable screening tool for development during the critical first five years of life.
- Through an interventional clinical trial, LI demonstrated an effective and tolerable alternative for ID treatment, while overcoming the usual adverse effects of conventional iron therapies.
- Growth and developmental outcomes improved significantly with early detection and treatment of ID with LI during the NAID stage than when the supplement was used after anemia emergence. Conversely, children with NAID who received a placebo showed further deterioration.

## Introduction

Iron deficiency is the leading nutritional deficiency in children in both developed and underdeveloped countries.^1^ Iron deficiency progresses in three stages, the first of which is characterized by a reduction in bone marrow iron reserves. With further depletion of iron reserves, erythropoiesis is compromised in the second stage. In these two stages, hemoglobin levels are still within normal values; however, serum ferritin levels are progressively decreasing, leading to non-anemic iron deficiency. Lastly, in the third stage, hemoglobin levels drop below age-appropriate thresholds with the development of iron deficiency anemia.^2^

Despite the impressive medical achievements, there are approximately 1.24 billion cases of iron deficiency anemia globally, with infants and preschool children having the highest prevalence. Furthermore, double this number lies under the tent of non-anemic iron deficiency.^3^

Several drawbacks of iron deficiency were documented in children, including delayed cognition, motor deficits, attention and memory deficits, visual and auditory disorders, in addition to poor academic performance.^4^ Current scientific evidence suggests that the age of onset, duration, and severity of iron deficiency, as well as the presence of anemia, may influence these cognitive and neurophysiological outcomes. Nevertheless, there is insufficient data to guarantee that iron supplementation could reverse these problems.^5,6^

Developmental screening is crucial for optimal development and early detection of any delay.^7^ A standard development screening tool should be cost-effective, simple, accurate, valid, reliable, culturally appropriate, as well as easy to administer and quick to use.^7,8^ The Ages and Stages Questionnaires—Third Edition (ASQ-3) have exceptional psychometric properties, including a test-retest reliability of 92%, a sensitivity of 87.4%, and a specificity of 95.7%. In addition, the validity of the Ages and Stages Questionnaires -3 has been examined in different cultures and communities and officially approved for use in both initial developmental screening and ongoing monitoring, making it a versatile tool for tracking a child’s developmental progress over time.^9^ Furthermore, Ages and Stages Questionnaires -3 encompasses five different areas of child development (communication, gross motor, fine motor, problem solving, and personal-social skills).^10^

There is little data on non-anemic iron deficiency screening and treatment, despite the wide spectrum of iron deficiency-related health hazards.^11^ Although the American Academy of Pediatrics (AAP) recommends screening of all children at the age of one year for iron deficiency anemia based on hemoglobin levels, currently there is no acknowledged guideline for non-anemic iron deficiency.

screening using the serum ferritin level, which corresponds proportionately with total body iron storage and has the highest specificity (98%) when acute inflammation and infection are absent. Consequently, iron deficiency is usually underestimated until iron deficiency anemia develops.^12,13^

Conventional oral iron supplementation used to treat iron deficiency has drawbacks, including poor tolerability and insufficient absorption (10–15%), which can result in non-compliance and treatment failure.^14^ Thanks to marvelous scientific achievements, novel iron preparations have been developed to counteract the drawbacks of conventional iron.^15^ Liposomal Iron (LI) stands first in these novel formulations.^16^ Liposomes are drug delivery vehicles that enclose a microdispersed granule, which is ferric pyrophosphate in the case of liposomal iron.^17^ Its average particle size is about 20–40 µm. Liposomal iron is absorbed in the intestine via five mechanisms. One of these is direct endocytosis at the M-cells in the small intestine, whereby liposomal iron bypasses the divalent metal ions transporter 1. Therefore, it is four times better absorbed compared to other conventional iron products. It is also stable for heat, salt, pH, and oxidation.^17,18^

Owing to contradictory results regarding the effects of iron supplementation on the reversal of developmental disorders and the lack of well-designed clinical trials to evaluate the efficacy of liposomal iron on the development of children with iron deficiency whether non-anemic iron deficiency or iron deficiency anemia,^19,20^ we aimed to investigate the efficacy of liposomal iron in the treatment of iron deficiency and its potential effects on children’s development and growth.

## Methods

### Study design

Our primary objective of this double-blind, placebo-controlled, randomized trial (DBPCT) was to determine the efficacy of liposomal iron therapy for 4 months in the developmental outcomes of children with non-anemic iron deficiency through the validated Ages and Stages Questionnaires -3. The secondary objective was to investigate whether early iron therapy administration in non-anemic iron deficiency has beneficial outcomes compared to iron deficiency with anemia regarding developmental scores, laboratory outcomes, and growth.

This trial was conducted from January 2022 up to March 2023. Before the start, approval was obtained under reference number 1/2022 PEDI 44 from the Institutional Research Board in Menoufia Faculty of Medicine, which works in accordance with the Declaration of Helsinki, and the trial was registered at the Pan African Clinical Trials Registry under number (PACTR202401875833105).

### Study population

A multistage random sampling technique was used to select ten nurseries from a stratified list of available nurseries at the time of study design. 674 apparently healthy children were screened for eligibility. The recruitment criteria were as follows: children aged 6 to 59 months, no history of consumption of iron supplements within the past 4 months, no history of blood transfusion, and no apparent signs of acute infections. We excluded children with any chronic disease, family history of hematological diseases, born prematurely or with low birth weight below 2500 grams, as well as children with evident neurological disabilities such as cerebral palsy, developmental delay, or fits.

433 children met all the inclusion criteria and were recruited for the study. All parents/guardians of the children in the ten nurseries attended a meeting during which study procedures and purposes were explained in detail, and written consent was obtained. Dietary recommendations were illustrated by a specialized dietitian for high-iron foods and foods that reduce iron absorption.^21^

Then we obtained a detailed history, thorough examination, and the socioeconomic standard (SES) was assessed using a scale developed by Fahmy et al.^22^

### Developmental assessment

All participants were screened by the Ages and Stages Questionnaires-3 by the appropriate questionnaire for each child’s age. The questionnaire consists of thirty items, six in each domain: communication, gross motor, fine motor, problem solving, and personal social.^9^ The questionnaire was translated into the Arabic language, and assessors were particularly trained to administer this questionnaire before data collection began. The Ages and Stages Questionnaires -3 in English and Arabic languages were added to the supplementary file 1 & 2.

Parents selected the appropriate response to each question, which is either yes (10 points), sometimes (5 points), or not yet (0 points). Then, scores from the five domains are added to determine the final score, which is a total of 300. After that, the child’s scores were compared to the cutoff points involved in the scoring sheet. Scores falling in the white area indicate the child is developing typically. If a child’s score falls within the gray zone, it is necessary to conduct additional testing in the future and closely monitor the child. Children whose scores fall within the black region have scored 2 standard deviations below the mean of their age-appropriate developmental levels. So that suggests a potential susceptibility to developmental delays.

### Anthropometric measurements

The child’s weight, length, and height were measured. If the child was below 2 years of age, weight for length was determined, and body mass index (BMI) was calculated for those who were over 2 years of age using the following formula: BMI = weight / (length)^2^^23^ Then, plotting of each measurement was done based on the latest Egyptian Z-score growth curves and their interpretation.^24^

### Laboratory investigations

A volume of five milliliters of blood was collected from each participant and divided into two tubes. Two milliliters were added to a tube containing EDTA for a complete blood count test conducted using the automated hematology analyzer (Swelab Alpha Basic, produced in Sudan), with a reference range for hemoglobin being 12–15 g/dl. For serum ferritin and C reactive protein (CRP), the other three milliliters were placed into plain tubes and left to clot, then serum was isolated from clotted blood by centrifugation for 10 minutes at 2000 Xg and a HIPRO-AFS/1 immunoassay was used to determine the level of serum ferritin, with reference range 30–400 µg/L. As ferritin is a positive acute phase protein, which can complicate the diagnosis of iron deficiency in the presence of concurrent inflammation, children with CRP levels above 5 mg/L were classified as positive CRP and were accordingly excluded from the study.^25^

### Study groups and intervention

**Fig. 1:**
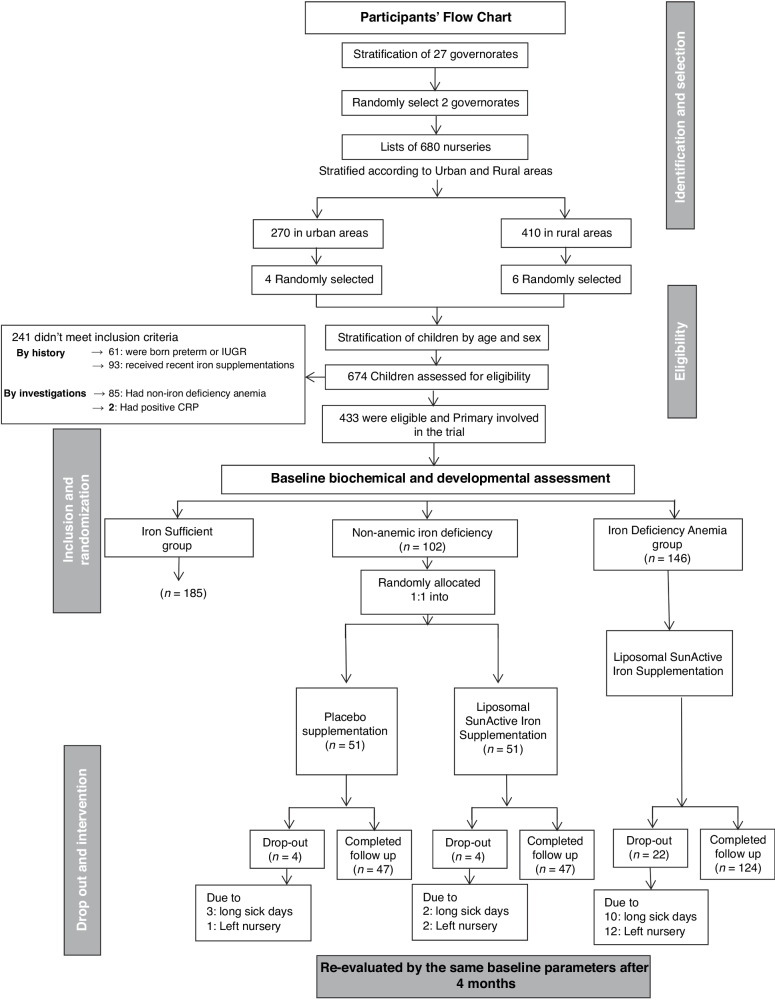
Flow chart of participants. Through a multistage random sampling technique, 674 apparently healthy children were selected from ten nurseries. 433 fulfilled all the inclusion criteria, 403 successfully completed the full structured study, and 30 children dropped out either due to noncompliance with therapy protocol or follow-up visits.

According to the WHO cutoff criteria for children less than five years, iron deficiency anemia is defined by hemoglobin level less than 11 g/dl, and a serum ferritin level less than 12 µg/L.^25,26^ Therefore, children with hemoglobin levels above 11 g/dl and serum ferritin levels above 12 µg/L were classified as iron sufficient and assigned to the iron sufficient group (*n* = 185) and were assigned as the control group. Children who have hemoglobin levels below 11 g/dl and serum ferritin levels below 12 µg/L were diagnosed with iron deficiency anemia and allocated to the iron deficiency anemia group (*n* = 146). Those with normal hemoglobin levels but serum ferritin levels less than 12 µg/L were classified as having non-anemic iron deficiency and were randomly assigned 1:1 to either the non-anemic iron deficiency placebo group (*n* = 51) or the non-anemic iron deficiency interventional group (*n* = 51). (Fig. 1).

To guarantee anonymity between the two comparator groups (non-anemic iron deficiency placebo and non-anemic iron deficiency interventional groups), all participants in the baseline analysis were assigned a unique personal code, which they used throughout the duration of the study. This code was kept confidential by an impartial monitoring board member until the data analyses were finished. Therefore, until the database is unlocked, participants, their families, and every member of the research team who is involved in gathering and evaluating outcome data are blind to the treatment allocation. A computer-generated list evolved a randomization schedule of permuted blocks stratified by sex and study sites to achieve balance.

An independent pharmacist prepared and pre-labeled packs (each containing the drug that matched the assigned trial code). Iron-supplemented groups (non-anemic iron deficiency interventional and iron deficiency anemia groups) received liposomal iron sachets (**FORTIFERRUM®, manufactured in Spain for A.E.I. 24**) once per day, six days per week. Each sachet contains 19.2 mg equivalent elemental iron pyrophosphate, which is reconstructed into 19.2 ml of water, forming a suspension of 1 mg per ml. According to the monthly updated weight, each participant received a dose of liposomal iron equal to 1.4 mg/kg/day.^16,27^ Children in the non-anemic iron deficiency placebo group received a daily dose from a placebo, which is a sachet of the same taste, color, and amount of liposomal iron with no active ingredients. Trained one field worker per nursery directly supervised the consumption of supplementation, and recorded compliance.

Adverse effects were monitored throughout the study by a questionnaire delivered to the parents of each child, which outlined the main adverse effects anticipated from iron therapy. Compliance was assessed by calculating the proportion of days on which individuals got the medication out of a maximum of 105 days.

### Assessment of interventional effect of liposomal iron

Four months after intervention, the final number of 403 children successfully completed the full structured study, while 30 children dropped out (Fig. 1). The estimated interventional effect was assessed for evaluation of the quantitative impact of liposomal iron intervention on laboratory, developmental and growth outcomes compared to baseline values expressed as the mean difference in each group. Furthermore, inter-group comparison was done to compare the differences in each stage, pre- and post-intervention, for detection if early treatment in the non-anemic iron deficiency stage is beneficial than treatment after the presence of iron deficiency anemia.

### Sample size calculation

The sample size was calculated using the SPSS computer program, based on previous studies evaluating iron therapy effectiveness and change in developmental scores between the baseline and 4 months after intervention.^19,28^ Using a confidence level of 95% and a statistical power of 80%, the smallest sample size of 45 children was required in each group. With an estimated 10% drop-out rate, we targeted having 51 children at the start in each comparative group.

### Statistical analysis

Data was analyzed using the IBM SPSS software package version 20.0*.* (Armonk, NY: IBM Corp). Qualitative data were described using numbers and percentages. The Kolmogorov-Smirnov test was used to verify the normality of the distribution. Quantitative data were described using mean ± standard deviation (SD) if normally distributed or median and interquartile range (IQR) if abnormally distributed. The Chi-square test was used to compare different groups for categorical variables. Monte Carlo correction was used for chi-square correction when more than 20% of the cells' expected count less than 5. For comparison between two periods, the Paired t-test was used for normally distributed quantitative variables and Mann Mann-Whitney test for abnormally distributed quantitative variables. The Kruskal-Wallis test was used for abnormally distributed quantitative variables, to compare more than two studied groups, and Post Hoc (Dunn’s multiple comparisons test) for pairwise comparisons. To evaluate the significance between the various stages, the McNemar Test was employed. The Spearman coefficient was used to determine the correlation between changes in serum ferritin and changes in development score. The Fisher Exact test was used for the analysis of contingency tables. *P* value ≤ 0.05 was considered significant.

## Results

The mean age of the participants was 28.49 ± 16.15 months and male participants represented 50.4%. Among the recruited sample (433), Iron deficiency was prevalent in 57.2% (33.7% iron deficiency anemia and 23.5% non-anemic iron deficiency). The Ages and Stages Questionnaires -3 development score was significantly higher in iron sufficient group than non-anemic iron deficiency and iron deficiency anemia groups, with the worst score in the iron deficiency anemia group. Regarding anthropometric measurements, the initial weight for age Z score, length/height for age Z score, weight for length and BMI Z score were significantly lower in non-anemic iron deficiency and iron deficiency anemia groups than the iron sufficient group. Baseline demographic characteristics, basic clinical and laboratory results are represented in (Table 1).

**Table 1:** Baseline demographic, clinical and laboratory data among the different studied groups.

	Total (*n* = 403)		Iron sufficient group (*n* = 185)		NAID placebo group (*n* = 47)		NAID interventional group (*n* = 47)		IDA group (*n* = 124)	
	No.	%	No.	%	No.	%	No.	%	No.	%
**Gender**										
Male	203	50.4	98	53.0	22	46.8	23	48.9	60	48.4
Female	200	49.6	87	47.0	25	53.2	24	51.1	64	51.6
**Age (months)**										
Mean ± SD.	28.49 ± 16.15		29.48 ± 15.19		24.17 ± 14.31		26.34 ± 15.88		27.38 ± 16.44	
**SES**										
Low	76	18.9	32	17.3	9	19.1	9	19.1	26	21.0
Middle	287	71.2	138	74.6	33	70.2	33	70.2	83	66.9
High	40	9.9	15	8.1	5	10.6	5	10.6	15	12.1
**Birth weight (kg)**										
Mean ± SD	3.45 ± 0.50		3.45 ± 0.50		3.30 ± 0.50		3.46 ± 0.53		3.49 ± 0.49	
**Gestational age (weeks)** Mean ± SD.	38.60 ± 1.04		38.65 ± 1.05		38.60 ± 1.01		38.57 ± 1.02		38.53 ± 1.03	
**Hemoglobin (g/dl)**(Mean ± SD)	11.15 ± 1.18		11.91 ± 0.62		11.64 ± 0.27		11.67 ± 0.39		9.63 ± 0.75	
**MCV (FL)** (Mean ± SD**)**	70.32 ± 13.35		83.68 ± 2.56		61.17 ± 6.65		61.23 ± 6.81		57.32 ± 6.10	
**Ferritin (µg/L)** Median (IQR)	16.83 ± 12.57		24.0 (19.0–31.0)		9.0 (7.50–10.0)		8.0 (7.0–9.0)		9.0 (7.0–10.0)	
**Developmental score** (Mean ± SD**)**	172 ± 27.63		201.3 ± 21.69		150.53 ± 24.39		151.9 ± 23.76		141.4 ± 27.02	
**Weight for age Z score** Median (IQR)	-0.89 ± 1.03		-0.50 (-0.90–0.30)		-1.50 (-1.80– -0.90)		-1.50 (-1.75–-0.80)		-1.50 (-2.0– -1.0)	
**Length/height for age Z score** Median (IQR)	-0.62 ± 0.99		-0.40 (-0.90–0.50)		-1.10 (-1.55–-0.40)		-1.10 (-1.50–-0.40)		-1.15 (-1.60 –-0.40)	
**Weight for length or BMI for age Z score** Median (IQR)	-0.92 ± 1.03		-0.40 (-1.10–0.30)		-1.50 (-1.85–-0.80)		-1.40 (-1.90–-0.90)		-1.60 (-2.05– -1.0)	

*NAID* non anemic iron deficiency, *IDA* iron deficiency anemia, *MCV* mean corpuscular volume.

*IQR* Inter quartile range, *SD* Standard deviation, *SES* socioeconomic standard.

### Laboratory parameters

The estimated intervention effect for hemoglobin in the non-anemic iron deficiency interventional group is +1.25 g/dL (95% CI: 1.07 – 1.43), meaning the iron supplementation led to a significant increase in hemoglobin levels. In contrast, the non-anemic iron deficiency placebo group showed an estimated effect of -0.18 g/dL, indicating a slight decline, which suggests that without intervention, hemoglobin levels may naturally decrease. Also, the post-therapy hemoglobin level was significantly higher in non-anemic iron deficiency interventional group compared to the iron deficiency anemia group (p3 < 0.001). (Table 2)

**Table 2:** Comparison between the different studied groups according to laboratory parameters.

Laboratory parameters	NAID placebo group (*n* = 47)	NAID interventional group (*n* = 47)	IDA group (*n* = 124)	P1	P2	P3
**Hemoglobin** (**g/dl)** (Mean ± SD**)**						
Before intervention	11.64 ± 0.27	11.67 ± 0.39	9.63 ± 0.75	<0.001**	0.995	<0.001**
After intervention	11.46 ± 0.28	12.92 ± 0.65	12.25 ± 0.58	<0.001**	<0.001**	<0.001**
**Estimated intervention effect**	−0.18 (−0.27–−0.10)	1.25 (1.07–1.43)	2.62 (2.48–2.75)			
**t (p**_**0**_**)**	4.415 (<0.001**)	13.915 (<0.001**)	39.039 (<0.001**)			
**MCV (FL)** (Mean ± SD**)**						
Before intervention	61.17 ± 6.65	61.23 ± 6.81	57.32 ± 6.10	<0.001**	0.963	<0.001**
After intervention	59.47 ± 7.20	81.74 ± 6.78	77.81 ± 6.21	<0.001**	<0.001**	<0.001**
**Estimated intervention effect**	−1.70 (−3.78–−0.38)	20.511 (17.73–23.29)	20.49 (18.95–22.03)			
**t (p**_**0**_**)**	1.650 (0.107)	14.63 (<0.001**)	26.2 (<0.001**)			
**Ferritin (µg/L)** Median (IQR)						
Before intervention	9.0 (7.50–10.0)	8.0 (7.0–9.0)	9.0 (7.0–10.0)	0. 335	0.275	0.180
After intervention	9.0 (7.0–9.0)	43.0 (35.50–46.5)	34.0 (32.0–43.0)	<0.001**	<0.001**	0.013^*^
**Estimated intervention effect**	0.34 (−0.70–1.38)	32.64 (29.42–35.86)	29.07 (27.27–30.87)			
**Z (p**_**0**_**)**	0.195 (0.845)	5.960 (<0.001**)	9.666 (<0.001**)			

*NAID* non-anemic iron deficiency, *IDA* iron deficiency anemia. *IQR* Interquartile range, *SD* Standard deviation, *Z* Wilcoxon signed ranks test, ***t*** *t*-test, *MCV* mean corpuscular volume.

p_0_: *p*-value for estimated intervention effect of each parameter by comparing effect of intervention/placebo versus before intervention/placebo values.

P1: *p*-value for intergroup comparison between the different studied groups before/ after intervention.

p2: *p*-value for comparing NAID placebo group and NAID interventional group before/ after intervention.

p_3:_*p*-value for comparing between NAID interventional group and IDA group before/ after intervention. *: Statistically significant at *p* ≤ 0.05.

** High statistically significant at *p* ≤ <0.001.

Regarding serum ferritin, no significant difference was observed in the non-anemic iron deficiency placebo group compared to the baseline level following 4 months of treatment, while the serum ferritin in the non-anemic iron deficiency interventional group and iron deficiency anemia group showed a substantial rise. A high statistical significance was found between the non-anemic iron deficiency interventional group and the non-anemic iron deficiency placebo group (p2 < 0.001) and between the non-anemic iron deficiency interventional group versus the iron deficiency anemia group 43.0 µg/L versus 34.0 µg/L (p3 = 0.013) (Table 2)

### Developmental scores

**Fig. 2:**
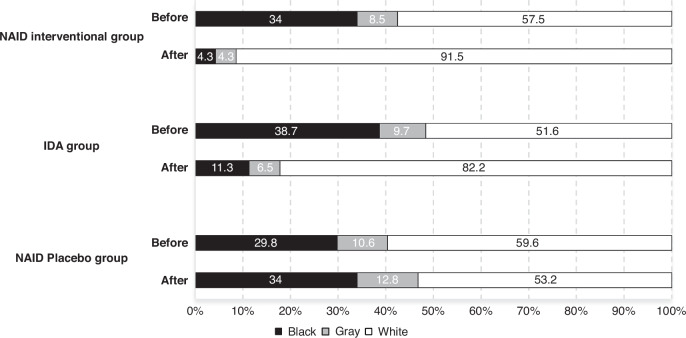
The Ages and Stages Questionnaires -3 before and after intervention in non-anemic iron deficiency (NAID) placebo group, non-anemic iron deficiency interventional group, and iron deficiency anemia (IDA) group. White areas represented those that were doing well, grey areas indicated the need for monitoring and intervention activities, and black areas meant that further assessment was recommended.

Regarding the impact of liposomal iron intervention on The Ages and Stages Questionnaire-3 interpretation, the percentage of children on the black zone reduced from 34% and 38.7% before intervention to 4.3% and 11.3% after 4 months of liposomal iron intervention in the non-anemic iron deficiency interventional group and iron deficiency anemia group, respectively. On the other hand, in the non-anemic iron deficiency placebo group the percentage of children on the black zone increased from 29.8% to 34%, indicating further deterioration with time (Fig. 2**)**. Regarding estimated intervention effect on developmental total scores, non-anemic iron deficiency interventional group showed significant superior performance with a mean difference of +29.57 compared to non-anemic iron deficiency placebo group, which had a mean difference only +0.96 (*P* < 0.001). Furthermore, the iron deficiency anemia group exhibited a noteworthy improvement of 33.63 points. However, the ultimate result was notably superior in the non-anemic iron deficiency interventional group compared to the iron deficiency anemia group (P3 < 0.001), with final scores of 181.5 and 175.0, respectively (Table 3).

**Table 3:** Comparison between the different study groups according to the developmental scores by the ages and stages Questionnaire -3 (ASQ- 3).

ASQ- 3	NAID Placebo group (*n* = 47)		NAID interventional group (*n* = 47)		IDA group (*n* = 124)		*P* value		
	No.	%	No.	%	No.	%			
**Before intervention**									
Black zone	14	29.8	16	34.0	48	38.7	^MC^p1 < 0.001*		
Gray zone	5	10.6	4	8.5	12	9.7			
White zone	28	59.6	27	57.5	64	51.6			
**After**									
Black zone	16	34.0	2	4.3	14	11.3	^MC^p1 < 0.001*		
Gray zone	6	12.8	2	4.3	8	6.5			
White zone	25	53.2	43	91.5	102	82.2			
^**MCN**^**p**_**0**_	0.625		<0.001*		<0.001*				
**Developmental total score** (Mean ± SD**)**									
Before intervention	150.53 ± 24.39		151.9 ± 23.76		141.4 ± 27.02		P1 < 0.001*	P2 = 0.992	P3 = 0.054
After intervention	151.5 ± 25.32		181.5 ± 21.99		175.0 ± 23.70		P1 < 0.001*	P2 < 0.001*	P3 < 0.001*
**Estimated intervention effect**	0.96 (-2.44–0.53)		29.57 (26.66–32.49)		33.63 (31.79–35.47)				
**t (p0)**	1.295 (0.202)		20.405 (<0.001*)		36.263 (<0.001*)				

*NAID* non anemic iron deficiency, *IDA* iron deficiency anemia.

*MC* Monte Carlo *McN* McNemar test, *t* t–test, *SD* Standard deviation.

p_0_: *p*-value for estimated intervention effect of each parameter by comparing the effect of intervention/placebo versus before intervention/placebo values.

P1: *p* value for intergroup comparison between the different studied groups before/ after intervention.

p2: *p* value for comparing the NAID placebo group and the NAID interventional group before/ after intervention.

p_3:_ *p* value for comparing between NAID interventional group and IDA group before/ after intervention. *High statistically significant at *p* ≤ <0.001.

white zone: refers to patient scores falling in the white area in ASQ-3, indicating that the child is developing typically.

Gray zone: refers to patient scores falling within the gray zone in ASQ-3, indicating that it is necessary to conduct additional testing in the future and closely monitor the child.

Black zone: refers to patient scores 2 standard deviations below the mean of their age-appropriate, thus falling within the black region developmental levels, suggesting a potential susceptibility to developmental delays.

**Fig. 3:**
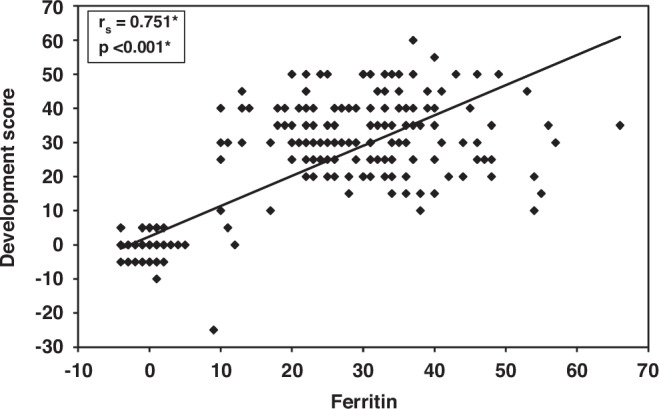
Correlation between serum ferritin level and the developmental score following the intervention.

Spearman correlation coefficient reveals a statistically significant positive correlation between serum ferritin level and changes in developmental score following intervention (rs = 0.751, *P* value < 0.001) (Fig. 3**)**.

### Growth

Both the non-anemic iron deficiency interventional group and the iron deficiency anemia group showed significant improvement in all anthropometric parameters (weight for age, length/height for age, and weight for length or BMI for age Z scores) after liposomal iron intervention (*p* < 0.001). Nevertheless, the non-anemic iron deficiency interventional group showed a significant superior improvement in weight and weight for length or BMI Z scores when compared with the iron deficiency anemia group (p3 < 0.001). Conversely, the non-anemic iron deficiency placebo group did not contribute significantly to the improvement in the three anthropometric measurements, as seen by their markedly lower Z scores compared to the non-anemic iron deficiency interventional group (*p* < 0.001) (Table 4).

**Table 4:** Comparison between the different studied groups according to anthropometric measures.

	NAID Placebo group (*n* = 47)	NAID interventional group (*n* = 47)	IDA group (*n* = 124)	P1	P2		P3
**Weight for age Z score :** Median (IQR)							
Before intervention	−1.50 (−1.80–−0.90)	−1.50 (−1.75–−0.80)	−1.50 (−2.0–−1.0)	<0.001**	0.960		0.331
After intervention	−1.60 (−1.90–−1.35)	−0.50 (−0.95–0.0)	−1.30 (−1.70–−0.50)	<0.001**	<0.001**		<0.001**
**Estimated intervention effect#**	0.12 (−0.15–0.39)	0.85 (0.51–1.19)	0.41 (0.21–0.61)				
**Z (p**_**0**_**)**	1.076 (0.282)	4.119 (<0.001**)	3.515 (<0.001**)				
**Length/height for age Z score :** Median (IQR)							
Before intervention	−1.10 (−1.55–−0.40)	−1.10 (−1.50–−0.40)	−1.15 (−1.60–−0.40)	<0.001**	0.922		0.823
After intervention	−1.20 (−1.55–−0.35)	−0.20 (−0.85 – 0.85)	−0.45 (−1.05–0.40)	<0.001**	<0.001**		0.093
**Estimated intervention effect**	0.01 (−0.05–0.07)	0.90 (0.52–1.29)	0.61 (0.44–0.77)				
**Z (p**_**0**_**)**	0.006 (0.995)	4.256 (<0.001**)	6.308 (<0.001**)				
**Weight for length or BMI for age Z score** Median (IQR)							
Before intervention	−1.50 (−1.85–−0.80)	-1.40 (-1.90–−0.90)	−1.60 (−2.05– −1.0)	<0.001**	0.930		0.445
After intervention	−1.60 (−1.90–−1.35)	−0.50 (−0.95–0.0)	−1.30 (−1.70–−0.50)	<0.001**	<0.001**		<0.001**
**Estimated intervention effect#**	0.10 (−0.19–0.38)	0.90 (0.56–1.23)	0.43 (0.22–0.63)				
**Z (p**_**0**_**)**	0.886 (0.376)	4.378 (<0.001**)	3.627 (<0.001**)				

*NAID* non anemic iron deficiency, *IDA* iron deficiency anemia, *IQR* Inter quartile range, *Z* Wilcoxon signed-rank test.

p_0_: *p* value for estimated intervention effect of each parameter by comparing the effect of intervention/placebo versus before intervention/placebo values.

P1: *p*-value for intergroup comparison between the different studied groups before/ after intervention.

p2: *p*-value for comparing NAID placebo group and NAID interventional group before/ after intervention.

p_3:_*p*-value for comparing between NAID interventional group and IDA group before/ after intervention.

**high statistically significant at *p* ≤ <0.00

### Compliance and adverse effects

We found that the non-anemic iron deficiency placebo, non-anemic iron deficiency interventional and iron deficiency anemia groups had relatively similar compliance days (93.9% ± 1.674, 93.2% ± 2.206, and 93.3% ± 1.9, respectively). Additionally, no serious adverse effects were observed in any of the groups. Constipation, loose stools, and darkening of the stool were observed in very close percentages among the three groups. (Table 5).

**Table 5:** Comparison between the different studied groups regarding drug compliance and side effects.

Participants‘ group	Compliance to drug (days) Mean ± S.D.	Side effects					
		Nil	Serious side effects	Teeth staining	Constipation	Loose stool	Black stool
**NAID placebo group (*n*** = **47)**	93.9 ± 1.674	45/47 (95.7%)	0/47 (0.0%)	0/47 (0. 0%)	1/47 (2.1%)	1/47 (2.1%)	0/47 (0.0%)
**NAID interventional group (*n*** = **47)**	93.2 ± 2.206	44/47 (93.6%)	0/ 47 (0.0%)	0/47 (0. 0%)	1/47 (2.1%)	1/47 (2.1%)	1\47 (2.1%)
**IDA group (*n*** = **124)**	93.3 ± 1.9	120/124 (96.77%)	0/124 (0.0%)	0/124 (0. 0%)	2/124 (1.6%)	2/124 (1.6%)	2/124 (1.6%)

*NAID* non anemic iron deficiency, *IDA* iron deficiency anemia.

## Discussion

One-third of children experience faltering in cognitive development by the time they reach preschool age.^29^ Research indicates that iron is crucial for various neurodevelopmental processes, particularly during early childhood, and has highlighted the significance of maintaining adequate iron levels during this critical period. However, the direct effects of iron deficiency on developmental outcomes remain uncertain.^30^ Thus, it is imperative to look at whether supplementing to address iron deficiency will improve developmental progress and to ascertain the best time to implement such interventions.

### Prevalence of iron deficiency anemia and non-anemic iron deficiency

The prevalence of iron deficiency anemia in our study was 33.7%, which matches the global prevalence of iron deficiency anemia (30%) as the most common nutritional deficiency of the population.^31^ Also, a previous study in Egypt showed that iron deficiency anemia prevalence among children aged 6-59 months ranged from 27% to 43% between 2014 and 2021.^32^ This prevalence could be considered a moderate risk according to the WHO’s public health grading criteria for iron deficiency anemia status in different communities.^33^

Regarding non-anemic iron deficiency, the prevalence was 23.5%, which is consistent with the global pooled prevalence of 17.95% among children under five years.^34^ However, non-anemic iron deficiency prevalence varies widely between nations, according to WHO statistics, as Africa reports 62.3% of iron deficiency cases, Southeast Asia 53.8%, while North America estimates 2.9% of cases.^35^ This variation may result from insufficient diagnosis and screening guidelines in addition that non-anemic iron deficiency is not considered a clinically suspicious condition, therefore falls outside the scope of clinical suspicion and is overlooked by physicians.^36^

### Iron deficiency and development

In our research, the non-anemic iron deficiency and iron deficiency anemia groups reported significantly lower developmental test scores compared to their iron-sufficient peers. Several recent studies have concluded that infants with iron deficiency, including those without anemia, have inferior performance on assessments measuring overall neurobehavioral development as well as gross motor, fine movement, and adaptability development compared to their counterparts with better iron levels.^37–39^ Also, Parkin et al. had investigated the correlation between serum ferritin and cognitive function in early childhood. Their findings indicated that greater serum ferritin values are linked to superior cognitive functions.^40^ This relationship was previously explained by the fact that iron deficiency impairs the function of iron-dependent enzymes crucial for neurotransmitter metabolism, DNA synthesis, and lipid synthesis in oligodendrocytes. Thus, brain myelination is slowed, neuro-synaptic structure becomes abnormal, and nerve growth factor expression is reduced.^41^

### Iron deficiency and growth

Iron deficiency is detrimental as it hampers cellular replication processes; specifically, the enzyme ribonucleotide reductase, which relies on iron, which is essential for initiating DNA synthesis.^42^ Furthermore, the implications of iron deficiency extend beyond cellular replication as it may also disrupt growth pathways that are influenced by insulin-like growth factor-1 (IGF-1) and IGF binding protein-1 -1 preventing cell proliferation with a negative impact on growth.^43^

In the same context, we observed that children with iron deficiency, regardless of the presence or absence of anemia, exhibited significantly lower mean Z-scores across all measured growth parameters, including weight, length/height, and BMI, than the iron sufficient group. Similarly, numerous observational studies have highlighted a concerning link between iron deficiency and hindered physical growth in children. Furthermore, malnourished children face a doubled risk of experiencing iron deficiency in contrast to their counterparts with a normal Z score.^44,45^

### Efficacy of liposomal iron intervention

As conventional iron supplementation for managing non-anemic iron deficiency was linked to a 30% occurrence of gastrointestinal side effects which decrease therapy adherence and compliance resulting in a high rate of dose reduction and treatment discontinuation, the inclusion of concealed iron in liposomal iron formulations leads to better absorption, avoiding unpleasant taste, and enhancing therapy adherences with lesser side effects reported in only 3.1% of children in a previous trial.^16,46^ Therefore, liposomal iron could be a promising alternative to conventional iron with good safety.

Liposomal iron was reported as an effective and safe substitute for intravenous iron gluconate for treating anemia in patients with chronic kidney disease.^17^ Also, additional research showed that liposomal iron was beneficial for non-iron-deficient cancer patients treated with epoetin alpha.^27^ However, because all previous data on liposomal iron in children were conducted on small sample sizes, limited to patients with mild iron deficiency anemia, and lacking its effects on iron storage replacement and hemoglobin stability after therapy, conclusions regarding the therapeutic effectiveness of liposomal iron for the treatment of children with iron deficiency anemia remain unclear.

Despite decades of research, there is still inconclusive evidence to determine the best course of action for treating iron deficiency in children. Heterogeneity of the study populations, especially the presence of anemia and iron status, compliance with the study interventions, and variations in the iron treatment’s dosage and duration, could all contribute to the inconsistent results across studies. Furthermore, there is a continuous argument for whether and how iron therapies enhance cognitive outcomes.^4^ Thus, we initiated this interventional double-blind, placebo-controlled trial to evaluate the efficacy of liposomal iron supplementation on iron deficiency and its effectiveness on the developmental performance and growth of children suffering from iron deficiency with or without anemia.

Hemoglobin and serum ferritin are the two most effective markers of iron therapy. Hemoglobin measurement is more commonly utilized since it is quick and affordable. Likewise, because of its broad availability and established high sensitivity and specificity for detecting iron deficiency in the absence of inflammation, serum ferritin level is an indicator of iron status.^47^ To guarantee full restoration of iron reserves in the body and to give sufficient time for discernible changes in development and growth, responses to our double-blind, placebo-controlled trial were evaluated four months later.

The placebo group’s hemoglobin levels significantly decreased while their serum ferritin remained unchanged; these results are consistent with previous research comparing iron supplementation and dietary recommendations for management of children with non-anemic iron deficiency.^20^ In contrast, our interventional liposomal iron groups revealed significant improvement in all hematological parameters with good compliance and few side effects (5.3%).

### Interventional effect of liposomal iron on development

Ages and Stages Questionnaires –3 developmental score improved significantly in liposomal iron interventional groups, with a highly significant correlation between the improvement in serum ferritin and the improvement in developmental score after. Nonetheless, the reversibility of developmental deficit was a point of conflict in many intervention studies. Idjradinata et al. demonstrated the beneficial impact of four months of oral iron therapy on the developmental test scores of young infants with iron deficiency.^48^ Conversely, Lozoff et al. found that iron therapy started after the onset of anemia did not reverse the negative neurological effects of anemia, even when an extensive course of iron therapy was taken.^49^ Further, a meta-analysis by Pasricha et al. revealed that most of the included studies found no significant improvement in cognitive development after iron supplementation.^4^

The significant disparities in the results of these studies can be attributed to several key factors, including the serum ferritin levels during screening, the anticipated adherence to the prescribed protocol, and that previous studies relied on conventional iron, which has many drawbacks that may affect the outcome. Furthermore, we chose to use the Ages and Stages Questionnaires –3 to evaluate developmental outcomes rather than using other developmental evaluations, such as the Belay scale, which has been criticized for potentially underestimating developmental delays. Moreover, in contrast to previous studies that used conventional iron treatments, our study is the first to use liposomal iron.

Although both liposomal iron interventional groups showed developmental score improvements, our results showed that the non-anemic iron deficiency group performed much better than the iron deficiency anemia group, with mean scores of 181.5 ± 21.99 and 175.0 ± 23.70, respectively (*P* value < 0.001). This difference highlights the value of early detection of iron deficiency and initiation of iron therapy before anemia develops in iron-deficient children to maximize their cognitive development.^50^ Also, compared to further deterioration without iron therapy in the non-anemic iron deficiency placebo group, our findings disprove the commonly accepted notion that dietary recommendations alone are adequate to sustain iron levels in non-anemic iron deficiency and emphasize the necessity of monitoring iron status in at-risk pediatric populations and early intervention.

### Interventional effect of liposomal iron on growth

In addition to the significant improvement in hematological measures and developmental scores, liposomal iron induced significant positive effects on all growth parameters. Many studies indicated that oral iron treatment was linked to a notably greater increase in both weight and height velocity when compared to placebo groups.^51,52^ Additionally, researchers observed a significant correlation between improved serum ferritin concentration and growth velocity as well as BMI in iron deficiency patients, highlighting a more pronounced effect than when patients were in iron deficiency anemia stage.^43^ This observation aligns with our study’s findings, where non-anemic iron-deficient interventional children exhibited improved growth metrics over those with iron-deficiency anemia.

Unfortunately, the impact of iron therapy on growth among children with iron deficiency remains contentious, and other systematic reviews assessing the role of iron supplementation on growth parameters in iron-deficient children reported no statistically significant positive outcomes. This conflict could stem from various factors, such as the selection of different age groups or the length of iron therapy administered. Furthermore, the lack of sufficient nutritional resources, particularly in developing nations, may hinder any potential growth improvements associated with iron supplementation.

### Points of strength

The strengths of our research are that this was the first study to evaluate the effectiveness of liposomal iron on iron deficiency through a double-blind, placebo-controlled randomized trial, which is the only way to definitively identify the effect of an intervention on the outcome. The interventional design effectively encompassed both the non-anemic iron deficiency and iron deficiency anemia groups to determine which stage is more advantageous for initiating iron therapy. Moreover, considering the significant consequences of iron deficiency in development and growth in children, we concurrently assessed them alongside biochemical results to determine their potential reversibility with iron therapy. Finally, we evaluated most social and demographic factors, which may have an additional impact on iron deficiency and the response to iron therapy.

### Limitations

The main limitations of our study are the heterogeneous nature of previous studies regarding developmental assessment, type and duration of iron therapy, and involvement of both anemic and non-anemic children, with resultant difficulty in comparison with them. In addition, studies on the efficacy of liposomal iron in children are scarce. Also, developmental assessment was obtained from parents who may introduce an inherent bias. Finally, follow-up over a longer period is needed to confirm the stability of these fruitful outcomes after discontinuation of the therapy.

Therefore, owing to the well-documented detrimental effects of iron deficiency on growth and development, we recommend screening for iron deficiency in children under five years by large prospective studies on diverse populations and in various medical conditions. Also, a standard guideline for diagnosis and management for non-anemic iron deficiency needs to be implemented.

## Conclusions

Both non-anemic iron deficiency and iron deficiency anemia had a negative impact on children’s developmental scores. Reversibility of regression is possible even if anemia occurs, but the outcome is better when intervention is done during the non-anemic iron deficiency stage. Liposomal iron therapy had influential imprints on development, growth, and biochemical improvement with high compliance and tolerability. Dietary modifications alone were not sufficient in non-anemic iron deficiency to improve development or growth, with potential for progression to iron deficiency anemia. Therefore, a lot of attention is needed to include early detection of iron deficiency by serum ferritin.

## Supplementary information

Supplementary File 1

Supplementary File 4

Supplementary File 3

## Data Availability

All data generated or analyzed during this study are included in this published article.
