# Supplementary material for: Interventional impact of liposomal iron on iron-deficient children developmental outcome: randomized, double-blind, placebo-controlled trial
Source: Pediatr Res. 2025 Jun 20;98(6):2228–39. doi: 10.1038/s41390-025-04204-9 (PMC12811114; doi:10.1038/s41390-025-04204-9)
Supplement: Supplementary file 2 — Supplementary File 4 [file 41390_2025_4204_MOESM2_ESM.pdf]

٦ شهور

السؤال	نعم	احيانا	ليس بعد
**مهارات الاتصال**			
١. هل ابنك بيعمل أصوات عالية لما بيتكلم أو يعيط؟	☐	☐	☐
٢. لما بتعمل لابنك أصوات مختلفة، هل ابنك بيعمل أصوات مختلفة؟	☐	☐	☐
٣. لو انت بعيد عن ابنك وندهت عليه، هل بيدور على مصدر الصوت؟	☐	☐	☐
٤. لما يبقى فيه صوت عالى، هل ابنك بيدير وشه يدور على مصدر الصوت؟	☐	☐	☐
٥. هل ابنك بيعمل أصوات زى دا، جا، كا، با؟	☐	☐	☐
٦. لو قلدت الأصوات اللى ابنك بيعملها، هل بيرجع يقلد الصوت تانى؟	☐	☐	☐
**النمو الحركى الاجمالى**			
١. لما ابنك يبقى نايم على ضهره، هل بيرفع رجله لدرجة يبقى شايفهم فيها؟	☐	☐	☐
٢. لما ابنك يبقى نايم على بطنه هل بيفرد دراعه الاتنين عشان يرفع صدره من السرير؟	☐	☐	☐
٣. هل ابنك بيتقلب من ظهره لبطنه ويشيل ايديه من تحته؟	☐	☐	☐
٤. لما تحط ابنك على الأرض، هل بيميل نفسه لقدام ويسند بايده على الأرض؟ "لو بيقعد لوحده" اختار نعم"	☐	☐	☐
٥. لو مسكت ايد ابنك عشان يتوازن، هل بيقدر يصلب طوله وهوا واقف؟	☐	☐	☐
٦. هل ابنك بياخد وضع الحبو؟ "بيسند على ايديه وركبه"؟	☐	☐	☐
**النمو الحركى الدقيق**			
١. هل ابنك بيمسك لعبة انت اديتهاله، بيلوح بيها ويبصلها، أو حتى بيحطها في بؤه لمدة دقيقة؟	☐	☐	☐
٢. هل بيحاول ابنك يوصل للعبة ويمسكها بايديه الاتنين في المرة الوحدة؟	☐	☐	☐
٣. هل ابنك بيحاول يمكس فتات عيش أو بسكوت بايده أو صوابعه؟ "لو ابنك بيمسك الحاجات الصغيرة زى البقوليات اختار نعم"	☐	☐	☐
٤. هل ابنك بيلقط لعبة صغيرة بايده وبيحوطها بصوابعه؟	☐	☐	☐
٥. هل بيحاول ابنك يمسك الفتافيت الصغيرة من الأرض بالابهام وباقى صوابعه وبيحرك ايده لقدام وورا حتى لو معرفش يجيبها في الآخر؟ "لو بيعرف يلقط الحاجات الصغيرة من الأرض اختار نعم"	☐	☐	☐
٦. هل ابنك ممكن يلقط اللعب الصغيرة من الأرض بايد وحدة؟	☐	☐	☐

حل المشكلات

١. لو فيه لعبة قدام ابنك، هل بيوصلها بايديه الاتنين؟ ☐ ☐ ☐

٢. لما ابنك يبقى نايم على ضهره، هل ممكن يحرك راسه يبص على لعبة وقعت منه؟ "لو بيمسك اللعبة اللى وقعت منه اختار نعم" ☐ ☐ ☐

٣. لما ابنك يبقى نايم على ضهره، هل بيحاول يجيب لعبة وقعت منه لو شايفها؟ ☐ ☐ ☐

٤. هل ابنك بيلقط لعبة من الأرض ويحطها في بؤه؟ ☐ ☐ ☐

٥. هل ابنك بيمرر لعبة من ايد للتانية رايح جاى؟ ☐ ☐ ☐

٦. هل ابنك بيخبط اللعبة في الترابيزة أو الأرض لفوق وتحت؟ ☐ ☐ ☐

الشخصية الاجتماعية

١. لما ابنك يبقى باصص للمراية، هل بيبتسم لنفسه أو بيعمل أصوات ؟ ☐ ☐ ☐

٢. هل ابنك بيتصرف بطريقة مختلفة لما بيشوف حد غريب؟ زى العياط، الابتعاد، التحليق في الشخص؟ ☐ ☐ ☐

٣. لما ابنك يبقى نايم على ضهره، هل بيمسك رجله بايده؟ ☐ ☐ ☐

٤. لما ابنك يبقى قدام المراية، هل بيحاول يوصلها ويسند عليها؟ ☐ ☐ ☐

٥. لما ابنك يبقى نايم على ضهره، هل بيحط رجله في بؤه؟ ☐ ☐ ☐

٦. هل ابنك بيحاول يوصل للعبة بعيدة عنه؟ ممكن يتقلب، أو يجى على بطنه؟ ☐ ☐ ☐

اسئلة عامة — نعم — لا

١. هل ابنك بيستخدم ايديه ورجليه الاتنين زى بعض؟ لو الاجابة لأ وضح؟ ☐ ☐

....

٢. لو ساعدت ابنك على الوقوف، هل بطن رجله بتبقى لامسه الأرض معظم الوقت؟ لو الاجابة لأ وضح؟ ☐ ☐

....

٣. هل عندك أى قلق ان ابنك هادى أوى أو مش بيعمل أصوات زى باقى الأطفال؟ لو الإجابة نعم وضح؟ ☐ ☐

....

٤. هل فيه تاريخ في العيلة سواء من الأب أو الأم للصم، أو مشاكل في السمع؟ لو الإجابة نعم وضح؟ ☐ ☐

....

٥. هل عندك قلق عن نظر ابنك؟ لو الإجابة نعم وضح؟ ☐ ☐

....

٦. هل ابنك عنده أى مشاكل صحية في آخر كام شهر؟ لو الإجابة نعم وضح؟ ☐ ☐

....

٧. هل عندك أى تساؤلات عن سلوكيات ابنك؟ لو الإجابة نعم وضح؟ ☐ ☐

....

٨. هل فيه أى حاجة ناحية ابنك تقلقك؟ لو الإجابة نعم وضح؟ ☐ ☐

....

٨ شهور

السؤال	نعم	احيانا	ليس بعد
**مهارات الاتصال**			
١. لو ندهت على ابنك وانت بعيد عنه، هل بيدور على مكان الصوت؟			
٢. هل ابنك بيدور على مصدر الصوت العالى؟			
٣. لو قلدت الأصوات اللى ابنك بيعملها، هل بيرجع يقلد الأصوات تانى؟			
٤. هل ابنك بيعمل أصوات زى دا، جا، كا، با؟			
٥. هل ابنك بيستجيب لنبرة صوتك المختلفة، وبيوقف لعب لو قولتله لأ؟			
٦. هل ابنك بيكرر الصوت مرتين زى بابا، دادا، جاجا؟ مش لازم الأصوات يبقى ليها معنى.			
**النمو الحركي الاجمالي**			
١. لما تحط ابنك على الأرض، هل بيميل نفسه لقدام، ويسند بايده على الأرض؟ "لو بيقعد لوحده اختار نعم"			
٢. هل ابنك بيتقلب من ضهره لبطنه ويشيل ايده من تحته؟			
٣. هل بياخد وضعية الحبو؟ بيسند على ايديه وركبه؟			
٤. لو مسكت ايد ابنك عشان يتوازن، هل بيقدر يصلب طوله وهو واقف؟			
٥. لو قعدت ابنك على الأرض، هل بيحاول يقعد وهو فارد نفسه لكذا دقيقة بدون ما يسند بايده؟			
٦. لما ابنك يقف وهوا ماسك أثاث البيت، هل بيقدر يقف وصدره وجسمه مش مسنودين على أثاث البيت؟			
**النمو الحركى الدقيق**			
١. هل ابنك بيحاول يمسك فتات عيش أو بسكوت بايده أو صوابعه؟ "لو ابنك بيمسك الحاجات الصغيرة زى البقوليات اختار نعم"			
٢. هل ابنك بيلقط لعبة صغيرة بايده وبيحوطها بصوابعه؟			
٣. هل بيحاول ابنك يمسك الفتافيت الصغيرة من الأرض بالابهام وباقى صوابعه وبيحرك ايده لقدام وورا حتى لو معرفش يجيبها في الآخر؟ "لو بيعرف يلقط الحاجات الصغيرة من الأرض اختار نعم"			
٤. هل ابنك ممكن يلقط اللعب الصغيرة من الأرض بايد وحدة؟			
٥. هل ابنك ممكن يلقط فرافيت العيش بالابهام وباقى الصوابع أو يحرك ايده لقدام وورا؟ "لو بيعرف يلقط الفرافيت الصغيرة اختار نعم"			
٦. هل ابنك يقدر يلقط اللعب الصغيرة بأطراف صوابعه؟ "لاحظ المسافة بين اللعبة وبطن ايده"			
**حل المشكلات**			
١. هل ابنك بيمسك اللعب ويحطها في بؤه؟			

٢. لو ابنك نايم على ضهره، هل بيحاول يجيب لعبة وقعت منه لو شايفها؟

٣. هل ابنك بيخبط اللعب في الترابيزة أو الأرض لفوق وتحت؟

٤. هل ابنك بيمرر لعبة من ايد للتانية، رايح جاى؟

٥. هل ممكن ابنك يمسك لعبة في كل ايد ويفضل ماسكهم لمدة دقيقة؟

٦. لو ابنك ماسك لعبة في ايده، هل بيخبطها في لعبة تانية موجودة على الترابيزة؟

الشخصية الاجتماعية

١. لما ابنك ينام على ضهره، هل بيمسك رجليه ويلعب بيها؟

٢. لما ابنك يبقى قدام المراية، هل بيحاول يوصلها ويسند عليها؟

٣. هل ابنك بيحاول يوصل للعبة بعيدة عنه؟ ممكن يتقلب أو يجى على بطنه؟

٤. لما ابنك يبقى نايم على ضهره، هل بيحط رجله في بؤه؟

٥. هل ابنك يشرب ميه أو عصير أو لبن من الكوباية وانت ماسكها؟

٦. هل ابنك ممكن يأكل نفسه بسكوتة؟

اسئلة عامة — نعم — لا

١. هل ابنك بيستخدم ايديه ورجليه الاتنين زى بعض؟ لو الاجابة لأ وضح؟

....

٢. لو ساعدت ابنك على الوقوف، هل بطن رجله بتبقى لامسه الأرض معظم الوقت؟ لو الاجابة لأ وضح؟

....

٣. هل عندك أى قلق ان ابنك هادى أوى أو مش بيعمل أصوات زى باقى الأطفال؟ لو الإجابة نعم وضح؟

....

٤. هل فيه تاريخ في العيلة سواء من الأب أو الأم للصم، أو مشاكل في السمع؟ لو الإجابة نعم وضح؟

....

٥. هل عندك قلق عن نظر ابنك؟ لو الإجابة نعم وضح؟

....

٦. هل ابنك عنده أى مشاكل صحية في آخر كام شهر؟ لو الإجابة نعم وضح؟

....

٧. هل عندك أى تساؤلات عن سلوكيات ابنك؟ لو الإجابة نعم وضح؟ ☐ ☐

....

٨. هل فيه أى حاجة ناحية ابنك تقلقك؟ لو الإجابة نعم وضح؟ ☐ ☐

....

٩ شهور

السؤال	نعم	احيانا	ليس بعد
**مهارات الاتصال**			
١. هل بيعمل أصوات زى دا، جا، كا، با؟	☐	☐	☐
٢. لو قلدت صوت ابنك، هل بيرجع يكرر نفس الصوت وراك؟	☐	☐	☐
٣. هل ابنك بيكرر نفس الصوت مرتين: بابا، دادا، جاجا؟	☐	☐	☐
٤. لو طلبت من ابنك يلعب لعبة، هل بيعرف يلعبها لوحده من غير ما توريه؟ على الأقل لعبة وحدة زى باى باى، يغمى عينه، يسقف؟	☐	☐	☐
٥. هل ابنك بينفذ أوامر بسيطة زى تعالا هنا، اديهانى، رجعها مكانها؟	☐	☐	☐
٦. هل ابنك بيقول ٣ كلمات زى ماما، داد، بابا؟ الكلمات لازم يكون مقصود بيها شخص أو شيء؟	☐	☐	☐
**النمو الحركى الاجمالى**			
١. لو مسكت ايد ابنك عشان يتوازن، هل بيقدر يصلب طوله وهو واقف؟	☐	☐	☐
٢. لو قعدت ابنك على الأرض، هل بيحاول يقعد وهو فارد نفسه، لكذا دقيقة بدون ما يسند بايده؟	☐	☐	☐
٣. لما ابنك يقف وهوا ماسك أثاث البيت، هل بيقدر يقف وصدره وجسمه مش مسنودين على أثاث البيت؟	☐	☐	☐
٤. لما ابنك يبقى واقف وهوا ماسك أثاث البيت ، هل يقدر ينزل يجيب حاجة من الأرض ويطلع تانى بدون ما يوقع؟	☐	☐	☐
٥. لما ابنك يبقى واقف وهوا ماسك أثاث البيت ، هل بيقدر يوطى نفسه بدون ما يوقع؟	☐	☐	☐
٦. هل ابنك يقدر يمشى وهوا ماسك أثاث البيت بايد وحدة؟	☐	☐	☐
**النمو الحركى الدقيق**			
١. هل ابنك ممكن يلقط اللعب الصغيرة من الأرض بايد وحدة؟	☐	☐	☐
٢. هل ابنك ممكن يلقط فرافيت العيش بالابهام وباقى الصوابع ويحرك ايده لقدام وورا؟ "لو بيعرف يلقط الفرافيت الصغيرة اختار نعم"	☐	☐	☐
٣. هل ابنك يقدر يلقط اللعب الصغيرة بأطرف صوابعه؟ "لاحظ المسافة بين اللعبة وبطن ايده"	☐	☐	☐
٤. هل ابنك ممكن يمسك حبل لعبة بالابهام وأول صباع؟ حتى لو بعد محاولات متكررة؟	☐	☐	☐
٥. هل ابنك ممكن يلقط الفرافيت بأطراف صوابعه؟ وايده تبقى مريحة على الأرض؟	☐	☐	☐
٦. هل ابنك ممكن يحط اللعب على الأرض بالراحة من غير ما تقع منه وبعدها يرفع ايده؟	☐	☐	☐

حل المشكلات

١. هل ابنك بيمرر لعبة من ايد للتانية، رايح جاى؟ ☐ ☐ ☐

٢. هل ممكن ابنك يمسك لعبة في كل ايد ويفضل ماسكهم لمدة دقيقة؟ ☐ ☐ ☐

٣. لو ابنك ماسك لعبة في ايده، هل بيخبطها في لعبة تانية موجودة على الترابيزة؟ ☐ ☐ ☐

٤. لما ابنك يبقى ماسك لعبة في كل ايد، هل بيسقف بيهم ويخبطهم في بعض؟ ☐ ☐ ☐

٥. هل ابنك بيحاول يمسك فرافيت عيش أو بسكوت موجودة جوه ازازة نضيفة؟ ☐ ☐ ☐

٦. لو خبيت لعبة قدام ابنك تحت ورقة أو هدوم، هل ممكن يلاقيها؟ ☐ ☐ ☐

الشخصية الاجتماعية

١. لما ابنك يبقى نايم على ضهره، هل بيحط رجله في بؤه؟ ☐ ☐ ☐

٢. هل ابنك بيشرب ميه أو عصير أو لبن من الكوباية وانت ماسكها؟ ☐ ☐ ☐

٣. هل ابنك ممكن يأكل نفسه بسكوتة؟ ☐ ☐ ☐

٤. لو مديت ايدك لابنك وطلبت منه اللعبة اللى في ايده، هل بيديهالك حتى لو مش حيسيبها من ايده؟ ☐ ☐ ☐

٥. هل ابنك بيساعدك لما تلبسه هدومه؟ مثلا يزق دراعه جوه الكم؟ ☐ ☐ ☐

٦. لو طلبت من ابنك اللعبة اللى في ايده، هل بيدهالك في ايدك؟ ☐ ☐ ☐

اسئلة عامة — نعم — لا

١. هل ابنك بيستخدم ايديه ورجليه الاتنين زى بعض؟ لو الاجابة لأ وضح؟ ☐ ☐

....

٢. لو ساعدت ابنك على الوقوف، هل بطن رجله بتبقى لامسه الأرض معظم الوقت؟ لو الاجابة لأ وضح؟ ☐ ☐

....

٣. هل عندك أى قلق ان ابنك هادى أوى أو مش بيعمل أصوات زى باقى الأطفال؟ لو الإجابة نعم وضح؟ ☐ ☐

....

٤. هل فيه تاريخ في العيلة سواء من الأب أو الأم للصم، أو مشاكل في السمع؟ لو الإجابة نعم وضح؟ ☐ ☐

....

٥. هل عندك قلق عن نظر ابنك؟ لو الإجابة نعم وضح؟ ☐ ☐

....

٦. هل ابنك عنده أى مشاكل صحية في آخر كام شهر؟ لو الإجابة نعم وضح؟ ☐ ☐

....

٧. هل عندك أى تساؤلات عن سلوكيات ابنك؟ لو الإجابة نعم وضح؟ ☐ ☐

....

٨. هل فيه أى حاجة ناحية ابنك تقلقك؟ لو الإجابة نعم وضح؟ ☐ ☐

....

١٠ شهور

السؤال	نعم	احيانا	ليس بعد
**مهارات الاتصال**			
١. هل بيعمل أصوات دا، جا، كا، با؟	☐	☐	☐
٢. لو قلدت صوت ابنك، هل بيردد وارك نفس الصوت؟	☐	☐	☐
٣. هل بيكرر نفس الصوت مرتين زى دادا، بابا، جاجا؟ مش لازم الأصوات يبقى ليها معنى.	☐	☐	☐
٤. هل ابنك ممكن يعمل لوحده باى باى، أو يسقف، أو يغمي عينيه؟	☐	☐	☐
٥. هل بينفذ الأوامر البسيطة؟ زى تعالا هنا، اديهالى، رجعها مكانها؟	☐	☐	☐
٦. هل ابنك بيقول ٣ كلمات: ماما، دادا، بابا؟ الكلمات الطفل يقصد بيها شيء أو شخص.	☐	☐	☐
**النمو الحركى الاجمالى**			
١. هل ابنك بيقف باتزان لما تمسك ايده؟	☐	☐	☐
٢. لو قعدت ابنك على الأرض، هل بيحاول يقعد وهو فارد نفسه لعدة دقايق بدون ما يسند بايده؟	☐	☐	☐
٣. لما ابنك يقف وهوا ماسك أثاث البيت، هل بيقدر يقف وصدره وجسمه مش مسنودين على أثاث البيت ؟	☐	☐	☐
٤. لما ابنك يبقى واقف وهو ماسك أثاث البيت ، هل يقدر ينزل يجيب حاجة من الأرض ويطلع تانى بدون ما يقع؟	☐	☐	☐
٥. لما ابنك يبقى واقف وهوا ماسك أثاث البيت ، هل بيقدر يوطى نفسه بدون ما يوقع؟	☐	☐	☐
٦. هل ابنك يقدر يمشى وهوا ماسك أثاث البيت بايد وحدة؟	☐	☐	☐
**النمو الحركى الدقيق**			
١. هل ابنك ممكن يلقط اللعب الصغيرة من الأرض بايد وحدة؟	☐	☐	☐
٢. هل ابنك ممكن يلقط فرافيت العيش بالابهام وباقى الصوابع ويحرك ايده لقدام وورا؟ "لو بيعرف يلقط الفرافيت الصغيرة اختار نعم"	☐	☐	☐
٣. هل ابنك يقدر يلقط اللعب الصغيرة بأطرف صوابعه؟ "لاحظ المسافة بين اللعبة وبطن ايده"	☐	☐	☐
٤. هل ابنك ممكن يمسك حبل لعبة بالابهام وأول صباع؟ حتى لو بعد محاولات متكررة؟	☐	☐	☐
٥. هل ابنك ممكن يلقط الفرافيت بأطراف صوابعه؟ وايده تبقى مريحة على الأرض؟	☐	☐	☐
٦. هل ابنك ممكن يحط اللعب على الأرض بالراحة من غير ما تقع منه وبعدها يرفع ايده؟	☐	☐	☐

حل المشكلات

١. هل ابنك بيمرر لعبة من ايد للتانية، رايح جاى؟ ☐ ☐ ☐

٢. هل ممكن ابنك يمسك لعبة في كل ايد ويفضل ماسكهم لمدة دقيقة؟ ☐ ☐ ☐

٣. لو ابنك ماسك لعبة في ايده، هل بيخبطها في لعبة تانية موجودة على الترابيزة؟ ☐ ☐ ☐

٤. لما ابنك يبقى ماسك لعبة في كل ايد، هل بيسقف بيهم ويخبطهم في بعض؟ ☐ ☐ ☐

٥. هل ابنك بيحاول يمسك فرافيت عيش أو بسكوت موجودة جوه ازازة نضيفة؟ ☐ ☐ ☐

٦. لو خبيت لعبة قدام ابنك تحت ورقة أو هدوم، هل ممكن يلاقيها؟ ☐ ☐ ☐

الشخصية الاجتماعية

١. لما ابنك يبقى نايم على ضهره، هل بيحط رجله في بؤه؟ ☐ ☐ ☐

٢. هل ابنك بيشرب ميه أو عصير أو لبن من الكوباية وانت ماسكها؟ ☐ ☐ ☐

٣. هل ابنك ممكن يأكل نفسه بسكوتة؟ ☐ ☐ ☐

٤. لو مديت ايدك لابنك وطلبت منه اللعبة اللى في ايده، هل بيديهالك حتى لو مش حيسيبها من ايده؟ ☐ ☐ ☐

٥. هل ابنك بيساعدك لما تلبسه هدومه؟ مثلا يزق دراعه جوه الكم؟ ☐ ☐ ☐

٦. لو طلبت من ابنك اللعبة اللى في ايده، هل بيدهالك في ايدك؟ ☐ ☐ ☐

**اسئلة عامة**	نعم	لا
١. هل ابنك بيستخدم ايديه ورجليه الاتنين زى بعض؟ لو الاجابة لأ وضح؟<br>..	☐	☐
٢. لو ساعدت ابنك على الوقوف، هل بطن رجله بتبقى لامسه الأرض معظم الوقت؟ لو الاجابة لأ وضح؟<br>..	☐	☐
٣. هل عندك أى قلق ان ابنك هادى أوى أو مش بيعمل أصوات زى باقى الأطفال؟ لو الإجابة نعم وضح؟<br>..	☐	☐
٤. هل فيه تاريخ في العيلة سواء من الأب أو الأم للصم، أو مشاكل في السمع؟ لو الإجابة نعم وضح؟<br>..	☐	☐
٥. هل عندك قلق عن نظر ابنك؟ لو الإجابة نعم وضح؟<br>..	☐	☐

٦. هل ابنك عنده أى مشاكل صحية في آخر كام شهر؟ لو الإجابة نعم وضح؟ ☐ ☐

....

٧. هل عندك أى تساؤلات عن سلوكيات ابنك؟ لو الإجابة نعم وضح؟ ☐ ☐

....

٨. هل فيه أى حاجة ناحية ابنك تقلقك؟ لو الإجابة نعم وضح؟ ☐ ☐

....

١٢ شهر

السؤال	نعم	احيانا	ليس بعد
**<u>مهارات الاتصال</u>**			
١. هل ابنك بيكرر نفس الصوت مرتين زى دادا، بابا، جاجا؟ مش لازم الأصوات تبقى ليها معنى.	☐	☐	☐
٢. هل ابنك ممكن يعمل لوحده باى باى، أو يسقف، أو يغمى عينيه؟	☐	☐	☐
٣. هل ابنك بينفذ الأوامر البسيطة؟ زى تعالا هنا، اديهالى، رجعها مكانها؟	☐	☐	☐
٤. هل ابنك بيقول ٣ كلمات: ماما، بابا، دادا؟ الكلمات الطفل يقصد بيها شيء أو شخص.	☐	☐	☐
٥. لو سألت ابنك، فين الكورة أو الشوز، هل بيبص عليها؟	☐	☐	☐
٦. لما ابنك بيحتاج حاجة، هل بيشاور عليها؟	☐	☐	☐
**<u>النمو الحركى الاجمالى</u>**			
١. لما ابنك يبقى واقف وهو ماسك أثاث البيت ، هل يقدر ينزل يجيب حاجة من الأرض ويطلع تانى بدون ما يوقع؟	☐	☐	☐
٢. هل ابنك ممكن يوطى وهوا ماسك أثاث البيت باتزان بدون ما يوقع؟	☐	☐	☐
٣. هل ممكن يمشى وهوا ساند على أثاث البيت بايد وحدة؟	☐	☐	☐
٤. لو مسكت ايد ابنك الاتنين عشان يمشى، هل ممكن ياخد خطوتين من غير ما يقع؟	☐	☐	☐
٥. لو مسكت ايد وحدة بس عشان تسند ابنك، هل ممكن يمشى خطوتين لقدام؟	☐	☐	☐
٦. هل ممكن ابنك يقف في نص الأوضة لوحده ويمشى خطوتين لقدام؟	☐	☐	☐
**<u>النمو الحركى الدقيق</u>**			
١. هل بيمسك ابنك حبل لعبة بالابهام والسبابة حتى لو بعد أكتر من محاولة؟	☐	☐	☐
٢. هل بيمسك ابنك فرافيت على الأرض بأطراف صوابعه، وباقى ايده مسنودة على الأرض؟	☐	☐	☐
٣. هل ممكن ابنك يحط لعبة على الأرض بالراحة من غير ما توقع منه، ويسيب ايده بعدها؟	☐	☐	☐
٤. هل ابنك بيمسك الفرافيت من على الترابيزة بأطراف صوابعه وايده مش مسنودة على الترابيزة؟	☐	☐	☐
٥. هل يقدر يحدف الكورة لقدام مع تحريك الدراع؟ "لو الكورة بتقع منه اختار ليس بعد".	☐	☐	☐
٦. هل ابنك بيساعدك في تقليب صفحات الكتاب؟	☐	☐	☐

حل المشكلات

١. لما ابنك بيمسك لعبة في كل ايد، هل بيخبطهم في بعض؟ ☐ ☐ ☐

٢. هل ابنك بيحاول يجيب فرافيت من جوة ازازة فاضية؟ زى ازازة عصير أو البيبرونة؟ ☐ ☐ ☐

٣. لو ابنك شافك وانت بتخبى لعبة تحت ورقة أو قماشة، هل يقدر يلاقيها؟ ☐ ☐ ☐

٤. لو حطيت لعبة صغيرة جوة علبة قدام ابنك، هل بيحاول يقلدك ويحط اللعبة جوة العلبة زيك؟ ☐ ☐ ☐

٥. هل ممكن ابنك يسقط اللعب الصغيرة جوة علبة وحدة ورا التانية؟ ☐ ☐ ☐

٦. لو شخبطت على ورقة بأقلام الشمع، هل بيقلدك ويشخبط زيك؟ "لو بيشخبط لوحده اختار نعم". ☐ ☐ ☐

الشخصية الاجتماعية

١. لو طلبت من ابنك يديك اللعبة اللى في ايده، هل بيناولها لك حتى لو مش حيسبهالك؟ ☐ ☐ ☐

٢. هل ابنك بيساعدك وانت بتلبسه وبيزق دراعه جوة الكم؟ ☐ ☐ ☐

٣. لو طلبت من ابنك اللعبة اللى في ايده، هل بيناولهالك ويسيبك تاخدها؟ ☐ ☐ ☐

٤. لما بتلبس ابنك، هل بيديلك رجله وانت بتلبسه الشراب أو الجزمة؟ ☐ ☐ ☐

٥. هل ابنك بيحدفلك الكورة، وبتلعبوا مع بعض بيها؟ ☐ ☐ ☐

٦. هل ابنك بيحضن لعبه الطرية زى العرايس أو الدباديب؟ ☐ ☐ ☐

اسئلة عامة نعم لا

١. هل ابنك بيستخدم ايديه ورجليه الاتنين زى بعض؟ لو الاجابة لأ وضح؟ ☐ ☐

....

٢. هل ابنك بيعمل أصوات كأنه بيقول كلمات ؟ لو الاجابة لا وضح ؟ ☐ ☐

....

٣. لو ساعدت ابنك على الوقوف، هل بطن رجله بتبقى لامسه الأرض معظم الوقت؟ لو الاجابة لأ وضح؟ ☐ ☐

....

٤. هل عندك أى قلق ان ابنك هادى أوى أو مش بيعمل أصوات زى باقى الأطفال؟ لو الإجابة نعم وضح؟ ☐ ☐

....

٥. هل فيه تاريخ في العيلة سواء من الأب أو الأم للصم، أو مشاكل في السمع؟ لو الإجابة نعم وضح؟

....

٦. هل عندك قلق عن نظر ابنك؟ لو الإجابة نعم وضح؟

....

٧. هل ابنك عنده أى مشاكل صحية في آخر كام شهر؟ لو الإجابة نعم وضح؟

....

٨. هل عندك أى تساؤلات عن سلوكيات ابنك؟ لو الإجابة نعم وضح؟

....

٩. هل فيه أى حاجة ناحية ابنك تقلقك؟ لو الإجابة نعم وضح؟

....

١٤ شهر

السؤال	نعم	احيانا	ليس بعد
**مهارات الاتصال**			
١. هل ابنك بيقول ٣ كلمات: ماما، بابا، دادا؟	☐	☐	☐
٢. لو ابنك عاوز حاجة، هل بيشاورلك عليها؟	☐	☐	☐
٣. هل ابنك بيهز راسه لو عايز يقول آه أو لأ؟	☐	☐	☐
٤. هل ابنك بيشاور على صورة في الكتاب، أو بيحاول يمسكها؟	☐	☐	☐
٥. هل ابنك ممكن يقول ٤ كلمات زيادة على بابا، ماما؟	☐	☐	☐
٦. لو طلبت من ابنك حاجة، هل ممكن يروح أوضة تانية عشان يجيبها؟ زى "فين الكورة، هاتلى الجاكيت، هات البطانية بتاعتك"	☐	☐	☐
**النمو الحركى الاجمالى**			
١. لو مسكت ايد ابنك الاتنين عشان يمشى، هل ممكن ياخد خطوتين من غير ما يقع؟	☐	☐	☐
٢. لو مسكت ايد وحدة بس عشان تسند ابنك، هل ممكن يمشى خطواتين لقدام؟	☐	☐	☐
٣. هل ابنك ممكن يقف في نص الأوضة لوحده، ويمشى خطوتين لقدام؟	☐	☐	☐
٤. هل ابنك ممكن يتشعلق أو يتسلق أثاث البيت والكراسى؟	☐	☐	☐
٥. هل ممكن ابنك يوطى عشان يجيب حاجة من الأرض ويقوم تانى بدون مساعدة؟	☐	☐	☐
٦. هل ابنك بيلف في الشقة مشى بدل ما يحبى؟	☐	☐	☐
**النمو الحركى الدقيق**			
١. هل ابنك بيمسك الفرافيت من على الترابيزة بأطراف صوابعه، وايده مش مسنودة على الترابيزة؟	☐	☐	☐
٢. هل يقدر يحدف الكورة لقدام مع تحريك الدراع؟ "لو الكورة بتقع منه اختار ليس بعد".	☐	☐	☐
٣. هل ابنك بيساعدك في تقليب صفحات الكتاب؟	☐	☐	☐
٤. هل ابنك بيركب اللعب فوق بعضها، مكعبات مثلا؟	☐	☐	☐
٥. هل ابنك ممكن يمسك قلم شمع ويعلم على الورقة؟	☐	☐	☐
٦. هل ابنك ممكن يركب ٣ مكعبات فوق بعض؟	☐	☐	☐
**حل المشكلات**			
١. لو حطيت لعبة صغيرة جوة علبة قدام ابنك، هل بيحاول يقلدك ويحط اللعبة جوة العلبة زيك؟	☐	☐	☐
٢. هل ممكن ابنك يسقط اللعب الصغيرة جوة علبة وحدة ورا التانية؟	☐	☐	☐

٣. لو شخبطت على ورقة بأقلام الشمع، هل بيقلدك ويشخبط زيك؟ "لو بيشخبط زيك اختار نعم".	☐	☐	☐
٤. هل ابنك ممكن يسقط فرافيت عيش أو بسكوت في ازازة فاضية، زى علب العصير أو الببرونة؟	☐	☐	☐
٥. هل ابنك ممكن يرمى كذا لعبة ورا بعض في بوكس أو طبق؟	☐	☐	☐
٦. لو وريت ابنك ازاى ممكن يقرب لعبة بمعلقة أو عصاية، هل ممكن يقلدك؟	☐	☐	☐

الشخصية الاجتماعية

١. لما بتلبس ابنك، هل بيديلك رجله وانت بتلبسه الشراب أو الجزمة؟	☐	☐	☐
٢. هل ابنك بيحدفلك الكورة، وبتلعبوا مع بعض بيها؟	☐	☐	☐
٣. هل ابنك بيحضن لعبه الطرية زى العرايس أو الدباديب؟	☐	☐	☐
٤. هل ابنك بيأكل نفسه بالمعلقة، حتى لو حيوقع شوية أكل على هدومه؟	☐	☐	☐
٥. هل ابنك بيساعدك في انه يقلع هدومه زى الشراب، الطقية، الشوز، الجوانتى؟	☐	☐	☐
٦. لو ابنك عايز يلفت نظرك لحاجة، هل يشد ايدك أو هدومك؟	☐	☐	☐

اسئلة عامة

	نعم	لا
١٠. هل ابنك بيستخدم ايديه ورجليه الاتنين زى بعض؟ لو الاجابة لأ وضح؟<br>..	☐	☐
١١. هل ابنك بيعمل أصوات كأنه بيقول كلمات ؟ لو الاجابة لا وضح ؟<br>..	☐	☐
١٢. لو ساعدت ابنك على الوقوف، هل بطن رجله بتبقى لامسه الأرض معظم الوقت؟ لو الاجابة لأ وضح؟<br>..	☐	☐
١٣. هل عندك أى قلق ان ابنك هادى أوى أو مش بيعمل أصوات زى باقى الأطفال؟ لو الإجابة نعم وضح؟<br>..	☐	☐
١٤. هل فيه تاريخ في العيلة سواء من الأب أو الأم للصم، أو مشاكل في السمع؟ لو الإجابة نعم وضح؟<br>..	☐	☐
١٥. هل عندك قلق عن نظر ابنك؟ لو الإجابة نعم وضح؟<br>..	☐	☐

١٦. هل ابنك عنده أى مشاكل صحية في آخر كام شهر؟ لو الإجابة نعم وضح؟ ☐ ☐

....

١٧. هل عندك أى تساؤلات عن سلوكيات ابنك؟ لو الإجابة نعم وضح؟ ☐ ☐

....

١٨. هل فيه أى حاجة ناحية ابنك تقلقك؟ لو الإجابة نعم وضح؟ ☐ ☐

....

16شهر

السؤال	نعم	احيانا	ليس بعد
**مهارات الاتصال**			
• هل ابنك بيشاور على صورة فى الكتاب أو بيحاول يمسكها بايده؟	☐	☐	☐
• هل ابنك بيقول على الأقل ٤ كلمات غير ماما وبابا؟	☐	☐	☐
• لما طفلك بيعوز حاجة معينة، هل بيشاورلك عليها؟	☐	☐	☐
• لما تطلب من ابنك حاجة، هل بيروح أوضة تانية عشان يجبهالك؟ "فين الكورة؟ هاتلى الجاكت، هات البطانية بتاعتك؟	☐	☐	☐
• هل ابنك بيكرر وراك جملة من كلمتين حتى لو مش بوضوح زى: ماما بتاكل، بابا بيلعب، ورح البيت؟	☐	☐	☐
• هل ابنك بيقول على الأقل ٨ كلمات غير ماما وبابا؟	☐	☐	☐
**النمو الحركى الاجمالى**			
• هل ابنك بيقف لوحده في نص الأوضة وبيحاول ياخد خطوات لقدام؟	☐	☐	☐
• هل ابنك ممكن يتشعلق أو يتسلق أثاث البيت أو الكراسى؟	☐	☐	☐
• هل ابنك بيقرفص عشان يلقط حاجة من الأرض ويقف تانى لوحده من غير أى مساعدة؟	☐	☐	☐
• هل ابنك بيتحرك في الشقة وهوا ماشى بدل ما يحبى؟	☐	☐	☐
• هل ابنك بيمشى كويس وبيوقع قليل؟	☐	☐	☐
• هل ابنك ممكن يطلع على الكرسى عشان يجيب حاجة من فوق زى لعبة مثلا أو يساعدك في المطبخ؟	☐	☐	☐
**النمو الحركى الدقيق**			
• هل ابنك بيقلب صفحات الكتاب معاك؟	☐	☐	☐
• هل ابنك بيرمى كورة صغيرة لقدام مع تحريك الدراع؟ "لو الكورة بتقع منه اختار ليس بعد".	☐	☐	☐
• هل ابنك ممكن يحط مكعب فوق مكعب أو لعبة فوق لعبة؟	☐	☐	☐
• هل ابنك ممكن يحط ٣ مكعبات أو لعب فوق بعضها لوحده؟	☐	☐	☐
• هل ابنك ممكن يشخبط على ورقة بقلم شمع أو رصاص أو حبر؟	☐	☐	☐
• هل ابنك ممكن يقلب صفحات الكتاب لوحده؟ حتى لو مجموعة صفحات مع بعض؟	☐	☐	☐
**حل المشكلات**			
• لما بتشخبط على ورقة بقلم شمع أو رصاص، هل ابنك بيحاول	☐	☐	☐

يقلدك ويشخبط زيك؟

- هل ابنك ممكن يرمى فرافيت بسكوت في ازازة شفافة صغيرة؟ زى ازازة العصير أو البيرونة؟
- هل ابنك ممكن يرمى اللعب ورا بعض في علبة أو بوكس؟
- هل ابنك ممكن يحاول يجيب لعبة بعيدة عنه بمعلقة أو عصاية بعد ما توريه ازاى؟
- هل ابنك ممكن يشخبط على الورقة بقلم الشمع بدون ما توريه ازاى؟
- لو بسكوتة وقعت جوة ازازة أو علبة، هل ابنك ممكن يقلب الازازة عشان يطلعها؟ "ممكن توريه ازاى".

الشخصية الاجتماعية

- هل ابنك بيأكل نفسه بالمعلقة حتى لو بيوقع شوية أكل على هدومه؟
- هل ابنك ممكن يقلع لنفسه الشراب، الشوز، الطقية، أو الجوانتيات؟
- هل ابنك ممكن يحضن اللعب الطرية زى العرايس والدباديب؟
- لما ابنك بيبص لنفسه في المراية، هل ممكن يدى لعبة لصورته في المراية؟
- هل ابنك بيحاول يلفت انتباهك أو يوريك حاجة بانه بشد ايدك أو هدومك؟
- هل ابنك بيجيلك لما بيحتاج منك مساعدة زى تشغيل لعبة، أو فتح غطا علبة؟

اسئلة عامة

نعم لا

- هل تعتقد ان ابنك بيسمع كويس ؟ لو الاجابة لأ وضح
- ....
- هل ابنك بيتكلم زي باقي الأطفال اللي في سنه؟ لو الاجابة لأ وضح
- هل تقدر تفهم معظم كلام ابنك؟ لو الاجابة لأ وضح؟

- هل ابنك بيمشي و يجري و يتسلق زي باقي الاطفال اللي في سنه؟ لوالاجابة لأ وضح؟ ▯ ▯
  -
- هل فيه تاريخ في العيلة سواء من الأب أو الأم للصم، أو مشاكل في السمع؟ لو الإجابة نعم وضح؟ ▯ ▯
  -
- هل عندك قلق عن نظر ابنك؟ لو الإجابة نعم وضح؟ ▯ ▯
  -
- هل ابنك عنده أى مشاكل صحية في آخر كام شهر؟ لو الإجابة نعم وضح؟ ▯ ▯
  -
- هل عندك أى تساؤلات عن سلوكيات ابنك؟ لو الإجابة نعم وضح؟ ▯ ▯
  -
- هل فيه أى حاجة ناحية ابنك تقلقك؟ لو الإجابة نعم وضح؟ ▯ ▯
  -

١٨ شهر

السؤال	نعم	احيانا	ليس بعد
**مهارات الاتصال**			
• لو ابنك احتاج حاجة، هل بيشاورلك عليها؟	☐	☐	☐
• لما تطلب من ابنك حاجة، هل بيروح أوضة تانية عشان يجبهالك؟ زى "فين الكورة؟ هات الجاكت، هات البطانية بتاعتك؟	☐	☐	☐
• هل ابنك بيقول على الأقل ٨ كلمات غير ماما وبابا؟	☐	☐	☐
• هل ابنك بيكرر وراك جملة من كلمتين حتى لو مش بوضوح؟ زى: ماما بتاكل، بابا بيلعب، روح البيت؟	☐	☐	☐
• بدون ما تورى ابنك الإجابة، هل ممكن يشاور على الصورة الصحيحة لما تسأله عليها؟ فين القطة؟ فين الكلب؟ "على الأقل يجاوب على صورة".	☐	☐	☐
• هل ابنك بيقول كلمتين أو تلاتة لأفكار مختلفة مع بعض؟ جملة وحدة؟ زى شوف الكب، ماما تعالى البيت، القطة مشيت. ما تحسبش الجمل اللى فيها نفس الكلمة أو المعنى زى: باى باى، طيب كويس، ايه ده؟	☐	☐	☐
**النمو الحركي الاجمالي**			
• هل ابنك بيقرفص عشان يلقط حاجة من الأرض؟ ويقف تانى لوحده من غير أى مساعدة؟	☐	☐	☐
• هل ابنك بيتحرك في الشقة وهو ماشى بدل ما يحبى؟	☐	☐	☐
• هل ابنك بيمشى كويس وبيوقع قليل؟	☐	☐	☐
• هل ابنك ممكن يتسلق على الكرسى عشان يوصل للحاجة اللى عاوزها؟ زى لعبة على الترابيزة أو يساعدك في المطبخ؟	☐	☐	☐
• هل ابنك بينزل السلالم لو مسكت ايده أو لما يسند على الحيطة أو سور السلم؟ ممكن تلاحظه في المحلات، أماكن اللعب أو البيت؟	☐	☐	☐

- لو وريت ابنك ازاى يشوط كورة كبيرة، هل بيحاول يشوطها برجله لقدام أو يمشى ناحيتها؟

النمو الحركى الدقيق

- هل ابنك بيرمى كورة صغيرة لقدام مع تحريك الدراع؟ "لو الكورة بتوقع منه اختار ليس بعد".
- هل ابنك ممكن يحط مكعب فوق مكعب أو لعبة فوق لعبة؟
- هل ابنك ممكن يشخبط على ورقة بقلم شمع أو رصاص أو حبر؟
- هل ابنك ممكن يحط ٣ مكعبات أو لعب فوق بعضها؟
- هل ابنك ممكن يقلب صفحات الكتاب لوحده؟ حتى لو مجموعة صفحات مع بعض؟
- هل ابنك بيعرف يمسك المعلقة ويوصلها لبوه ومش بيوقع الأكل؟

حل المشكلات

- هل ابنك ممكن يرمى اللعب ورا بعض في علبة أو بوكس؟
- هل ابنك ممكن يجيب لعبة بعيدة عنه بمعلقة أو عصاية بعد ما توريه ازاى؟
- لو بسكوته وقعت جوه ازازة أو علبة، هل ابنك ممكن يقلب الازازة عشان يطلعها؟ "ممكن توريه ازاى".
- هل ابنك ممكن يشخبط على الورقة بقلم الشمع أو حبر أو رصاص بدون ما توريه ازاى؟
- لو وريت ابنك ازاى يرسم خط من أول الورقة لآخرها بقلم شمع أو حبر أو رصاص، هل ممكن يقلدك ويرسم خط مستقيم في أى اتجاه؟ "لو لسه بيشخبط بس اختار ليس بعد".
- لو بسكوته وقعت جوه ازازة أو علبة، هل ابنك ممكن يقلب الازازة عشان يطلعها؟ "بدون ما توريه ازاى".

الشخصية الاجتماعية

- لما ابنك بيبص على نفسه في المراية، هل ممكن يدى لعبة لصورته في المراية؟
- هل ابنك بيحضن اللعب الطرية زى العرايس والدباديب؟
- هل ابنك بيحاول يلفت انتباهك أو يوريك حاجة بانه يشد ايدك أو هدومك؟
- هل ابنك بيجيلك لما يحتاج مساعدة، زى انه يشغل لعبة أو يفتح

غطا علبة؟

- هل ابنك بيعرف يشرب من الكوباية لوحده ويرجعها تانى على الترابيزة بدون ما يوقع كتير؟
- هل ابنك بيقلدك في تصرفاتك؟ زى مسح بقعة، الكنس، الحلاقة، أو تسريح الشعر؟

اسئلة عامة نعم لا

١. هل تعتقد ان ابنك بيسمع كويس ؟ لو الإجابة لأ وضح

....

٢. هل ابنك بيتكلم زي باقي الأطفال اللي في سنه؟ لو الإجابة لأ وضح

....

٣. هل تقدر تفهم معظم كلام ابنك؟ لو الإجابة لأ وضح؟

....

٤. هل ابنك بيمشي و يجري و يتسلق زي باقي الاطفال اللي في سنه؟ لو الإجابة لأ وضح؟

....

٥. هل فيه تاريخ في العيلة سواء من الأب أو الأم للصم، أو مشاكل في السمع؟ لو الإجابة نعم وضح؟

....

٦. هل عندك قلق عن نظر ابنك؟ لو الإجابة نعم وضح؟

....

٧. هل ابنك عنده أى مشاكل صحية في آخر كام شهر؟ لو الإجابة نعم وضح؟

....

٨. هل عندك أى تساؤلات عن سلوكيات ابنك؟ لو الإجابة نعم وضح؟

....

٩. هل فيه أى حاجة ناحية ابنك تقلقك؟ لو الإجابة نعم وضح؟

....

٢٠ شهر

السؤال	نعم	احيانا	ليس بعد
**مهارات الاتصال**			
• هل ابنك بيكرر وراك جملة من كلمتين، حتى لو مش بوضوح، زى ماما بتاكل، بابا بيلعب، روح البيت؟			
• هل ابنك بيقول على الأقل ٨ كلمات غير ماما وبابا؟			
• بدون ما تورى ابنك الاجابة، هل ممكن يشاور على الصورة الصحيحة لما تسأله عليها؟ زى فين القطة، فين الكلب، على الأقل يجاوب على صورة وحدة؟			
• لو شاورت على صورة كورة، قطة، كوباية، طقية، وسألت ابنك ايه ده؟ هل بيجاوب على الأقل صورة وحدة صحيحة؟			
• بدون ما تساعد ابنك، هل ابنك ممكن ينفذ على الأقل ٣ من الأوامر دى؟ حط اللعبة على الترابيزة، دور على الجاكت، اقفل الباب، امسك ايدى، هاتلى الفوطة، هات الكتاب؟			
• هل ابنك بيقول كلمتين أو تلاتة لأفكار مختلفة مع بعض في جملة وحدة، زى شوف الكلب، ماما تعالى البيت، القطة مشيت، "متحسبش الجمل اللى فيها نفس الكلمة أو المعنى، زى باى باى، طيب كويس، ايه ده؟			
**النمو الحركي الاجمالي**			
• هل ابنك ممكن يتسلق على الكرسى عشان يوصل للحاجة اللى عاوزها؟ زى لعبة على الترابيزة أو يساعدك في المطبخ؟			
• هل ابنك بيمشى كويس ويوقع قليل؟			

- هل ابنك بينزل السلالم لو مسكت ايده أو لما يسند على الحيطة أو سور السلم؟ ممكن تلاحظه في المحلات، أماكن اللعب أو البيت؟
- لو وريت ابنك ازاى يشوط كورة كبيرة، هل بيحاول يشوطها برجله لقدام أو يمشى ناحيتها؟
- هل ابنك بيجرى كويس إلى حد كبيرة، وبيقف لوحده من غير ما يتكعبل أو يوقع؟
- هل ابنك ممكن يطلع أو ينزل سلمتين لوحده؟ ممكن يسند على سور السلم أو الحيطة؟

<u>النمو الحركى الدقيق</u>

- هل ابنك ممكن يشخبط على ورقة بقلم شمع أو رصاص أو حبر؟
- هل ابنك ممكن يحط مكعب فوق مكعب أو لعبة فوق لعبة؟
- هل ابنك ممكن يقلب صفحات الكتاب لوحده؟ حتى لو مجموعة صفحات مع بعض؟
- هل ابنك بيعرف يمسك المعلقة ويوصلها لبؤه ومش بيوقع الأكل؟
- هل ابنك بيعرف يحط ٦ مكعبات فوق بعض لوحده؟
- هل ابنك بيعرف يحرك ايده بطريقة دائرية؟ زى يفتح أوكرة الباب؟ أو يشغل زمبلك لعبة؟ أو يفتح ويقفل غطا علبة؟

<u>حل المشكلات</u>

- هل ابنك ممكن يشخبط على الورقة بقلم الشمع أو بحبر أو برصاص بدون ما توريه ازاى؟
- لو وريت ابنك ازاى يرسم خط من أول الورقة لآخرها بقلم شمع أو حبر أو رصاص، هل ممكن يقلدك ويرسم خط مستقيم في أى اتجاه؟ "لو لسة بيشخبط بس اختار ليس بعد".
- لو عملت حاجة من الأفعال دى، هل ابنك ممكن يقلد على الأقل حاجة وحدة؟ افتح واقفل بؤك، شد ودنك، بربش بعينيك، انفخ خدودك؟
- لو اديت لابنك ازازة مقلوبة أو معلقة أو قلم معكوس، هل بيقلبهم بحيث يستخدمهم صح؟
- لو رصيت ٤ مكعبات جنب بعض وعملت بيهم صف، هل ممكن ابنك يقلدك على الأقل بمكعبين؟
- لو ابنك عاير يجيب حاجة ومش طايلها، هل بيروح يجيب كرسى أو بوكس عشان يقف عليه؟ زى يجيب لعبة من على الترابيزة أو

- يساعدك في المطبخ؟

الشخصية الاجتماعية

- هل ابنك بيأكل نفسه بالمعلقة حتى لو بيوقع شوية أكل على هدومه؟
- هل ابنك بيحاول يلفت انتباهك أو يوريك حاجة بانه يشد ايدك أو هدومك؟
- هل ابنك بيعرف يشرب من الكوباية لوحده ويرجعها تانى على الترابيزة بدون ما يقوع كتير؟
- هل ابنك بيقلدك في تصرفاتك؟ زى مسح بقعة، الكنس، الحلاقة أو تسريح الشعر؟
- لما ابنك بيلعب مع العرايس أو الدباديب بتاعته، هل بيهزها، بيأكلها، بيغيرلها البامبرز، بينيمها على السرير؟
- هل ابنك بياكل بالشوكة؟

اسئلة عامة نعم لا

١. هل تعتقد ان ابنك بيسمع كويس ؟ لو الإجابة لأ وضح

....

٢. هل ابنك بيتكلم زي باقي الأطفال اللي في سنه؟ لو الإجابة لأ وضح؟

....

٣. هل تقدر تفهم معظم كلام ابنك؟ لو الإجابة لأ وضح؟

....

٤. هل ابنك بيمشي و يجري و يتسلق زي باقي الاطفال اللي في سنه؟ لو الإجابة لأ وضح؟

....

٥. هل فيه تاريخ في العيلة سواء من الأب أو الأم للصم، أو مشاكل في السمع؟ لو الإجابة نعم وضح؟

....

٦. هل عندك قلق عن نظر ابنك؟ لو الإجابة نعم وضح؟

....

٧. **هل ابنك عنده أى مشاكل صحية في آخر كام شهر؟ لو الإجابة نعم وضح؟**

....

٨. **هل عندك أى تساؤلات عن سلوكيات ابنك؟ لو الإجابة نعم وضح؟**

....

٩. **هل فيه أى حاجة ناحية ابنك تقلقك؟ لو الإجابة نعم وضح؟**

....

٢٢ شهر

السؤال	نعم	احيانا	ليس بعد
**مهارات الاتصال**			
لو شاورت على صورة كورة، قطة، كوباية، طقية، وسألت ابنك ايه ده؟ هل بيجاوب على الأقل على صورة وحدة صحيحة؟	☐	☐	☐
بدون ما تساعد ابنك، هل ابنك ممكن ينفذ على الأقل ٣ من الأوامر دى؟ حط اللعبة على الترابيزة، دور على الجاكيت، اقفل الباب، امسك ايدى، هاتلى الفوطة، هات الكتاب؟	☐	☐	☐
لو طلبت من ابنك يشاور على أجزاء جسمه، زى الأنف، العينين، الشعر، الودن، هل بيشاور على ٧ أجزاء على الأقل؟ "ممكن يشاور على نفسه أو على باباه أو على لعبة"، "لو شاور على ٣ بس اختار أحيانا".	☐	☐	☐
هل ابنك بيقول على الأقل ١٥ كلمة بالإضافة إلى ماما وبابا؟	☐	☐	☐
هل ابنك بيستخدم على الأقل كلمتين من الكلمات دى بطريقة صحيحة: أنا، بتاعتى، أنت؟	☐	☐	☐
هل ابنك بيقول كلمتين أو تلاتة لأفكار مختلفة مع بعض في جملة وحدة، زى شوف الكلب، ماما تعالى البيت، القطة مشيت، "متحسبش الجمل اللى نفس الكلمة أو المعنى زى باى باى، طيب كويس، ايه ده"؟	☐	☐	☐
**النمو الحركى الاجمالى**			
لو وريت ابنك ازاى يشوط كورة كبيرة، هل بيحاول يشوطها برجله لقدام أو بيمشى ناحيتها؟	☐	☐	☐
هل ابنك بيجرى كويس إلى حد كبير، وبيقف لوحده من غير ما يتكعبل أو يوقع؟	☐	☐	☐
هل ابنك بينزل السلالم لو مسكت ايده أو لما يسند على الحيطة أو سور السلم؟ ممكن تلاحظه في المحلات، أماكن اللعب أو البيت؟	☐	☐	☐
هل ابنك ممكن يطلع أو ينزل سلمتين لوحده؟ ممكن يسند على سور السلم أو الحيطة؟	☐	☐	☐
هل ابنك بيعرف ينط برجليه الاتنين ويبعد عن الأرض؟	☐	☐	☐
هل ابنك بيعرف يشوط الكورة لقدام بدون ما يبقى ساند على حاجة؟	☐	☐	☐

النمو الحركى الدقيق

- هل ابنك بيعرف يمسك المعلقة ويوصلها لبؤه ومش بيوقع الأكل؟
- هل ابنك بيعرف يحط ٦ مكعبات فوق بعض لوحده؟
- هل ابنك بيعرف يحرك ايده بطريقة دائرية؟ زى يفتح أوكرة الباب؟ أو يشغل زمبلك لعبة؟ أو يفتح ويقفل غطا علبة؟
- هل ابنك ممكن يقلب صفحات الكتاب لوحده؟ حتى لو مجموعة صفحات مع بعض؟
- هل ابنك ممكن يفتح ويقفل مفتاح النور؟
- هل ممكن ابنك يدخل حبل أو رباط شوز جوه خرز كبير أو مكرونه؟

حل المشكلات

- هل ابنك ممكن يشخبط على الورقة بقلم الشمع أو بحبر أو رصاص بدون ما توريه ازاى؟
- لو رصيت ٤ مكعبات جنب بعض، وعملت بيهم صف، هل ممكن ابنك يقلدك على الأقل بمكعبين؟
- هل ابنك بيستخدم الأشياء بصورة مختلفة، زى يحط كوباية على ودنه على أنها تلفون، أو علبة على راسه على انها طقية؟ هل بيتسخدم اللعب عشان يقلب الأكل؟
- لو وريت ابنك ازاى يرسم خط من أول الورقة لآخرها، بقلم شمع أو حبر أو رصاص، هل ممكن يقلدك ويرسم خط مستقيم في اى اتجاه؟ "لو لسة بيشخبط بس اختار ليس بعد".
- لو بسكوتة وقعت جوه ازازة أو علبة، هل ابنك ممكن يقلب الازازة عشان يطلعها؟ "بدون ما توريه ازاى"؟
- لو اديت لابنك ازازة مقلوبة أو معلقة أو قلم معكوس، هل بيقلبهم بحيث يستخدمهم صح؟

الشخصية الاجتماعية

- هل ابنك بيقلدك في تصرفاتك؟ زى مسح بقعة، الكنس، الحلاقة، أو تسريح الشعر؟
- لو عملت حاجة من الأفعال دى، هل ابنك ممكن يقلد على الأقل حاجة وحدة؟ افتح واقفل بؤك، شد ودنك، بربش بعينيك، انفخ خدودك؟
- هل ابنك بياكل بالشوكة؟
- هل ابنك بيعرف يشرب من الكوباية لوحده ويرجعها تانى على

الترابيزة بدون ما يوقع كتير؟

- لما ابنك بيلعب مع العرايس أو الدباديب بتاعته، هل بيهزها، بيأكلها، بيغيرلها البامبرز، بينيمها على السرير؟
- هل ابنك بيزق عربية أو لعبة بعجلات بين العفش وبيرجع بيها لورا؟

اسئلة عامة

	نعم	لا
١. هل تعتقد ان ابنك بيسمع كويس ؟ لو الإجابة لأ وضح	☐	☐
٢. هل ابنك بيتكلم زي باقي الأطفال اللي في سنه؟ لو الإجابة لأ وضح؟	☐	☐
٣. هل تقدر تفهم معظم كلام ابنك؟ لو الإجابة لأ وضح؟	☐	☐
٤. هل ابنك بيمشي و يجري و يتسلق زي باقي الاطفال اللي في سنه؟ لو الإجابة لأ وضح؟	☐	☐
٥. هل فيه تاريخ في العيلة سواء من الأب أو الأم للصم، أو مشاكل في السمع؟ لو الإجابة نعم وضح؟	☐	☐
٦. هل عندك قلق عن نظر ابنك؟ لو الإجابة نعم وضح؟	☐	☐
٧. هل ابنك عنده أى مشاكل صحية في آخر كام شهر؟ لو الإجابة نعم وضح؟	☐	☐
٨. هل عندك أى تساؤلات عن سلوكيات ابنك؟ لو الإجابة نعم وضح؟	☐	☐
٩. هل فيه أى حاجة ناحية ابنك تقلقك؟ لو الإجابة نعم وضح؟	☐	☐

٢٤ شهر

السؤال	نعم	احيانا	ليس بعد
**مهارات الاتصال**			
• هل ابنك بيشاور على الصورة الصحيحة لما تسأله بدون ما تساعده؟ "فين القطة، فين الكلب"؟ "لازم يتعرف على صورة وحدة على الأقل".	☐	☐	☐
• هل ابنك بيكرر وراك جملة من كلمتين؟ حتى لو مش بوضوح؟ زى ماما بتاكل، بابا بيلعب، روح البيت.	☐	☐	☐
• بدون ما تساعد ابنك، هل ابنك ممكن ينفذ على الأقل ٣ من الأوامر دى؟ حط اللعبة على الترابيزة، دور على الجاكيت، اقفل الباب، امسك ايدى، هاتلى الفوطة، هات الكتاب؟	☐	☐	☐
• لو شاورت على صورة كورة، قطة، كوباية، طقية، وسألت ابنك ايه ده؟ هل بيجاوب على صورة وحدة صحيحة على الأقل؟	☐	☐	☐
• هل ابنك بيقول كلمتين أو تلاتة لأفكار مختلفة مع بعض في جملة وحدة، زى شوف الكلب، ماما تعالى البيت، القطة مشيت؟ "متحسبش الجمل اللى فيها نفس الكلمة أو المعنى، زى باى باى، طيب كويس، ايه ده"؟	☐	☐	☐
• هل ابنك بيستخدم الكلمات دى بطريقة صحيحة زى أنا، تباعتى، إنت؟	☐	☐	☐
**النمو الحركي الاجمالي**			
• هل ابنك بينزل السلالم لو مسكت ايده أو لما يسند على الحيطة أو سور السلم؟ ممكن تلاحظه في المحلات، أماكن اللعب، أو البيت؟	☐	☐	☐
• لو وريت ابنك ازاى يشوط كورة كبيرة، هل بيحاول يشوطها برجله لقدام أو يمشى ناحيتها؟	☐	☐	☐
• هل ابنك ممكن يطلع أو ينزل سلمتين لوحده؟ ممكن يسند على سور السلم أو الحيطة؟	☐	☐	☐

- هل ابنك بيجرى كويس إلى حد كبير، وبيقف لوحده من غير ما يتكعبل أو يوقع؟
- هل ابنك بيعرف ينط برجليه الاتنين ويبعد عن الأرض؟
- هل ابنك بيعرف يشوط الكورة لقدام بدون ما يبقى ساند على حاجة؟

النمو الحركى الدقيق

- هل ابنك بيعرف يمسك المعلقة ويوصلها لبؤه ومش بيوقع الأكل؟
- هل ابنك ممكن يقلب صفحات الكتاب لوحده؟ حتى لو مجموعة صفحات مع بعض؟
- هل ابنك بيعرف يحرك ايده بطريقة دائرية؟ زى يفتح أوكرة الباب، أو يشغل زمبلك لعبة، أو يفتح ويقفل غطا علبة؟
- هل ممكن يفتح ويقفل مفتاح النور؟
- هل ابنك بيعرف يحط ٧ مكعبات أو لعب فوق بعض؟
- هل ممكن ابنك يدخل حبل أو رباط شوز جوه خرز كبير أو مكرونة؟

حل المشكلات

- لو وريت ابنك ازاى يرسم خط من أول الورقة لآخرها، بقلم شمع أو حبر أو رصاص، هل ممكن يقلدك ويرسم خط مستقيم في اى اتجاه؟ "لو لسة بيشخبط بس اختار ليس بعد".
- لو بسكوتة وقعت جوه ازازة أو علبة، هل ابنك ممكن يقلب الازازة عشان يطلعها؟ "بدون ما توريه ازاى".
- هل ابنك بيستخدم الأشياء بصورة مختلفة، زى يحط كوباية على ودنه على انها تلفون، أو علبة على راسه على انها طقية، هل بيستخدم اللعب عشان يقلب الاكل؟
- هل ابنك بيرص الحاجات في مكانها؟ مثلا بيحط لعبته في رف اللعب، أو البطانية على السرير، أو الأطباق في المطبخ؟
- لو ابنك عايز يجيب حاجة ومش طايلها، هل بيروح يجيب كرسى أو بوكس عشان يقف عليه؟ زى يجيب لعبة من على الترابيزة أو يساعدك في المطبخ؟
- لو ابنك شافك وانت بترص ٤ مكعبات أو عربيات جنب بعض صف واحد، هل ممكن يقلدك؟

الشخصية الاجتماعية

- هل ابنك بيعرف يشرب من الكوباية لوحده ويرجعها تانى على

الترابيزة بدون ما يوقع كتير؟

- هل ابنك بيقلدك في تصرفاتك، زى مسح بقعة، الكنس، الحلاقة، أو تسريح الشعر؟
- هل ابنك بياكل بالشوكة؟
- لما ابنك بيلعب مع العرايس أو الدباديب بتاعته، هل بيهزها، بيأكلها، بيغيرلها البامبرز، بينيمها على السرير؟
- هل ابنك بيزق عربية أو لعبة بعجلات بين العفش وبيرجع بيها لورا؟
- هل ابنك بيستخدم "أنا" أكتر من اسمه؟ مثلا أنا بعملها بدل أحمد بيعملها؟

اسئلة عامة نعم لا

١. هل تعتقد ان ابنك بيسمع كويس ؟ لو الإجابة لأ وضح

....

٢. هل ابنك بيتكلم زي باقي الأطفال اللي في سنه؟ لو الإجابة لأ وضح؟

....

٣. هل تقدر تفهم معظم كلام ابنك؟ لو الإجابة لأ وضح؟

....

٤. هل ابنك بيمشي و يجري و يتسلق زي باقي الاطفال اللي في سنه؟ لو الإجابة لأ وضح؟

....

٥. هل فيه تاريخ في العيلة سواء من الأب أو الأم للصم، أو مشاكل في السمع؟ لو الإجابة نعم وضح؟

....

٦. هل عندك قلق عن نظر ابنك؟ لو الإجابة نعم وضح؟

....

٧. هل ابنك عنده أى مشاكل صحية في آخر كام شهر؟ لو الإجابة نعم وضح؟

....

٨. هل عندك أى تساؤلات عن سلوكيات ابنك؟ لو الإجابة نعم وضح؟

....

٩. هل فيه أى حاجة ناحية ابنك تقلقك؟ لو الإجابة نعم وضح؟

....

٢٧ شهر

السؤال	نعم	احيانا	ليس بعد
**مهارات الاتصال**			
بدون ما تساعد ابنك، هل ابنك ممكن ينفذ على الأقل ٣ من الأوامر دى؟ حط اللعبة على الترابيزة، دور على الجاكيت، اقفل الباب، امسك ايدى، هاتلى الفوطة، هات الكتاب؟	☐	☐	☐
لو شاورت على صورة كورة، قطة، كوباية، طقية، وسألت ابنك ايه ده؟ هل بيجاوب على صورة وحدة صحيحة على الأقل؟	☐	☐	☐
لو طلبت من ابنك يشاور على أجزاء جسمه، زى الأنف، العينين، الشعر، الودان، هل بيشاور على ٧ أجزاء على الأقل؟ "ممكن يشاور على نفسه، أو على باباه، أو على لعبة" "لو شاور على ٣ بس اختار أحيانا".	☐	☐	☐
هل ابنك بيستخدم على الأقل كلمتين من الكلمات دى بطريقة صحيحة: أنا، بتاعتى، إنت؟	☐	☐	☐
هل ابنك بيكون جمل من ٣ أو ٤ كلمات؟ ادينى مثال؟	☐	☐	☐
بدون ما تساعد ابنك، اسأله: حط الكتاب على الترابيزة، حط الشوز تحت الكرسى، هل ممكن ينفذ الأوامر دى بطريقة صحيحة؟	☐	☐	☐
**النمو الحركى الاجمالى**			
هل ابنك ممكن يطلع أو ينزل سلمتين لوحده؟ ممكن يسند على سور السلم أو الحيطة؟	☐	☐	☐
هل ابنك بيجرى كويس إلى حد كبير، ويقف لوحده من غير ما يتكعبل أو يوقع؟	☐	☐	☐
هل ابنك بيعرف ينط برجليه الاتنين ويبعد عن الأرض؟	☐	☐	☐
هل ابنك بيعرف يشوط الكورة لقدام بدون ما يبقى ساند على	☐	☐	☐

حاجة؟

- هل ابنك ممكن ينط لقدام مسافة ٥سم ورجليه الاتنين بعيدة عن الأرض؟
- هل ابنك ممكن يطلع السلم وهوا مستخدم رجل وحدة على كل سلمة؟ "الرجل اليمين على سلمة، والرجل الشمال على سلمة تانية"، ممكن يسند على سور السلم أو الحيطة.

النمو الحركى الدقيق

- هل ابنك بيعرف يحرك ايده بطريقة دائرية، زى يفتح أوكرة الباب، أو يشغل زمبلك لعبة، أو يفتح ويقفل غطا لعبة؟
- هل ابنك ممكن يفتح ويقفل مفتاح النور؟
- لو وريت ابنك ازاى يرسم خط من أول الورقة لآخرها، بقلم شمع أو حبر أو رصاص، هل ممكن يقلدك ويرسم خط مستقيم بصورة "عمودية"؟
- هل ابنك بيعرف يحط ٧ مكعبات أو لعب فوق بعض؟
- هل ممكن ابنك يدخل حبل أو رباط شوز جوه خرز كبير أو مكرونة؟
- لو وريت ابنك ازاى يرسم خط من أول الورقة لآخرها بقلم شمع أو حبر أو رصاص، هل ممكن يقلدك ويرسم خط مستقيم بصورة أفقية؟

حل المشكلات

- هل ابنك بيستخدم الأشياء بصورة مختلفة، زى يحط كوباية على ودنه على انها تلفون، أو علبة على راسه على انها طقية، هل بيستخدم اللعب عشان يقلب الأكل؟
- هل ابنك بيرص الحاجات في مكانها؟ مثلا بيحط لعبته في رف اللعب، أو البطانية على السرير، أو الأطباق في المبطخ؟
- لو سألت ابنك وهوا باصص لنفسه في المراية، فين .... "اسم ابنك"، هل بيشاور على صورته في المراية؟
- لو ابنك عايز يجيب حاجة ومش طايلها، هل بيروح يجيب كرسى أو بوكس عشان يقف عليه، زى يجيب لعبة من على الترابيزة، أو يساعدك في المطبخ؟
- لو ابنك شافك وانت بترص ٤ مكعبات أو عربيات جنب بعض صف واحد، هل ممكن يقلدك؟
- لو شاورت على الرسمة وسألت ابنك ايه ده؟ هل ممكن يقول

كلمة يقصد بيها شخص أو حاجة؟ زى: رجل التلج، ولد، راجل، بنت، بابا"، سجل رد ابنك.

الشخصية الاجتماعية

- لو عملت حاجة من الأفعال دى، هل ابنك ممكن يقلد على الأقل حاجة وحدة؟ افتح واقفل بؤك، شد ودنك، بربش بعينيك، انفخ خدوك.
- هل ابنك بياكل بالشوكة؟
- لما ابنك بيلعب مع العرايس، أو الدباديب بتاعته، هل بيهزها، بيأكلها، بيغيرلها البامبرز، بينيمها على السرير؟
- هل ابنك بيزق عربية أو لعبة بعجلات بين العفش وبيرجع بيها لورا؟
- هل ابنك بيستخدم "أنا" أكثر من اسمه، مثلا: أنا بعملها بدل أحمد بيعملها؟
- هل ابنك ممكن يلبس الجاكيت أو التيشرت لوحده؟

اسئلة عامة — نعم — لا

- هل تعتقد ان ابنك بيسمع كويس ؟ لو الإجابة لأ وضح
- ....
......
- هل ابنك بيتكلم زي باقي الأطفال اللي في سنه؟ لو الإجابة لأ وضح؟
- هل تقدر تفهم معظم كلام ابنك؟ لو الإجابة لأ وضح؟
- هل ابنك بيمشي و يجري و يتسلق زي باقي الاطفال اللي في سنه؟ لو الإجابة لأ وضح؟
- هل فيه تاريخ في العيلة سواء من الأب أو الأم للصم، أو مشاكل في السمع؟ لو الإجابة نعم وضح؟
- هل عندك قلق عن نظر ابنك؟ لو الإجابة نعم وضح؟

- هل ابنك عنده أى مشاكل صحية في آخر كام شهر؟ لو الإجابة نعم وضح؟
- هل عندك أى تساؤلات عن سلوكيات ابنك؟ لو الإجابة نعم وضح؟
- هل فيه أى حاجة ناحية ابنك تقلقك؟ لو الإجابة نعم وضح؟
- ....

٣٠ شهر

السؤال	نعم	احيانا	ليس بعد
**مهارات الاتصال**			
لو شاورت على صورة كورة، قطة، كوباية، طقية، وسألت ابنك ايه ده؟ هل بيجاوب على صورة وحدة صحيحة على الأقل؟			
بدون ما تساعد ابنك، هل ابنك ممكن ينفذ على الأقل ٣ من الأوامر دى؟ حط اللعبة على الترابيزة، دور على الجاكيت، اقفل الباب، امسك ايدى، هاتلى الفوطة، هات الكتاب؟			
لو طلبت من ابنك يشاور على أجزاء جسمه، زى الأنف، العينين، الشعر، الودان، هل بيشاور على ٧ أجزاء على الأقل؟ "ممكن يشاور على نفسه، أو على باباه، أو على لعبة" "لو شاور على ٣ بس اختار أحيانا".			
هل ابنك بيكون جمل من ٣ أو ٤ كلمات؟ ادينى مثال؟			
بدون ما تساعد ابنك، اسأله: حط الكتاب على الترابيزة، حط الشوز تحت الكرسى، هل ممكن ينفذ الأوامر دى بطريقة صحيحة؟			
لو وريت ابنك صورة في كتاب وسألته: الولد بيعمل ايه؟ هل بيجاوب بالفعل الصحيح؟ بياكل، بيجرى، .. ؟			
**النمو الحركي الاجمالي**			
هل ابنك بيجرى كويس إلى حد كبير، ويقف لوحده من غير ما يتكعبل أو يوقع؟			
هل ابنك ممكن يطلع أو ينزل سلمتين لوحده، ممكن يسند على سور السلم أو الحيطة؟			

- هل ابنك بيعرف يشوط الكورة لقدام بدون ما يبقى ساند على حاجة؟
- هل ابنك بيعرف ينط برجليه الاتنين ويبعد عن الأرض؟
- هل ابنك ممكن يطلع السلم وهوا مستخدم رجل وحدة على كل سلمة؟
- هل ابنك ممكن يقف على رجل وحدة لمدة ثانية بدون ما يمسك في حاجة؟

النمو الحركى الدقيق

- هل ابنك بيعرف يحرك ايده بطريقة دائرية، زى يفتح أوكرة الباب، أو يشغل زمبلك لعبة، أو يفتح ويقفل غطا لعبة؟
- لو وريت ابنك ازاى يرسم خط من أول الورقة لآخرها، بقلم شمع أو حبر أو رصاص، هل ممكن يقلدك ويرسم خط مستقيم بصورة "عمودية"؟
- هل ممكن ابنك يدخل حبل أو رباط شوز جوه خرز كبير أو مكرونة؟
- لو وريت ابنك ازاى يرسم خط من أول الورقة لآخرها، بقلم شمع أو حبر أو رصاص، هل ممكن يقلدك ويرسم خط مستقيم بصورة "أفقية"؟
- لو رسمت دايرة قدام ابنك، هل ممكن يقلدك ويرسم دايرة لوحده؟
- هل ابنك ممكن يقلب صفحات الكتاب صفحة بصفحة؟

حل المشكلات

- وابنك باصص لنفسه في المراية، اسأله: فين .... "اسم ابنك"، هل بيشاور على صورته في المراية؟
- لو ابنك عايز يجيب حاجة ومش طايلها، هل بيروح يجيب كرسى أو بوكس عشان يقف عليه، زى يجيب لعبة من على الترابيزة، أو يساعدك في المطبخ؟
- لو ابنك شافك وانت بترص ٤ مكعبات أو عربيات جنب بعض صف واحد، هل ممكن يقلدك؟
- لو شاورت على الرسمة وسألت ابنك ايه ده؟ هل ممكن يقول كلمة يقصد بيها شخص أو حاجة؟ زى رجل التلج، ولد، راجل، بنت، بابا، سجل رد ابنك.
- لو قلت لابنك قول "سبعة – تلاتة" أو "تمانية – اتنين" هل ابنك بيكرر وراك الرقمين بنفس الترتيب؟

- لو ابنك رسم رسمة بسيطة وسألته ايه ده؟ هل بيقولك رسم ايه؟

الشخصية الاجتماعية

- لو عملت حاجة من الأفعال دى، هل ابنك ممكن يقلد على الأقل حاجة وحدة؟ افتح واقفل بؤك، شد ودنك، بربش بعينيك، انفخ خدودك.
- هل ابنك بيأكل نفسه بالمعلقة بدون ما يوقع كتير؟
- هل ابنك بيزق عربية أو لعبة بعجلات بين العفش وبيرجع بيها لورا؟
- هل ابنك ممكن يلبس الجاكيت أو التيشرت لوحده؟
- وانت بتلبس ابنك النبطلون، هل ممكن يشده لفوق لحد وسطه؟
- لما ابنك يبقى باصص لنفسه في المراية وسألته مين في المراية؟ هل بيقولك أنا أو يقول اسمه؟

اسئلة عامة نعم لا

١. هل تعتقد ان ابنك بيسمع كويس ؟ لو الإجابة لأ وضح

....

٢. هل ابنك بيتكلم زي باقي الأطفال اللي في سنه؟ لو الإجابة لأ وضح؟

....

٣. هل تقدر تفهم معظم كلام ابنك؟ لو الإجابة لأ وضح؟

....

٤. هل ابنك بيمشي و يجري و يتسلق زي باقي الاطفال اللي في سنه؟ لو الإجابة لأ وضح؟

....

٥. هل فيه تاريخ في العيلة سواء من الأب أو الأم للصم، أو مشاكل في السمع؟ لو الإجابة نعم وضح؟

....

٦. هل عندك قلق عن نظر ابنك؟ لو الإجابة نعم وضح؟

....

٧. هل ابنك عنده أى مشاكل صحية في آخر كام شهر؟ لو الإجابة نعم وضح؟ ☐ ☐

....

٨. هل عندك أى تساؤلات عن سلوكيات ابنك؟ لو الإجابة نعم وضح؟ ☐ ☐

....

٩. هل فيه أى حاجة ناحية ابنك تقلقك؟ لو الإجابة نعم وضح؟ ☐ ☐

....

٣٣ شهر

السؤال	نعم	احيانا	ليس بعد
**مهارات الاتصال**			
• ظهر لطفلك كيف يتحرك سوستة على معطف لأعلى ولأسفل ، وقل ، " انظر ، هذا يرتفع لأعلى ولأسفل."ضع سوستة في المنتصف واطلب من طفلك تحريك السحاب لأسفل. أعد السحاب إلى المنتصف واطلب من طفلك تحريك سوستة لأعلى. افعل ذلك عدة مرات			
• ٦. عندما تسأل, "ما هو اسمك?"هل يقول طفلك اسمه الأول أو لقبه؟			
• لو طلبت من ابنك يشاور على أجزاء جسمه، زى الأنف، العينين، الشعر، الودان، هل بيشاور على ٧ أجزاء على الأقل؟ "ممكن يشاور على نفسه، أو على باباه، أو على لعبة" "لو شاور على ٣ بس اختار أحيانا".			
• هل ابنك بيكون جمل من ٣ أو ٤ كلمات؟ ادينى مثال؟			
• بدون ما تساعد ابنك، اسأله: حط الكتاب على الترابيزة، حط الشوز تحت الكرسى، هل ممكن ينفذ الأوامر دى بطريقة صحيحة؟			
• لو وريت ابنك صورة في كتاب وسألته: الولد بيعمل ايه؟ هل بيجاوب بالفعل الصحيح؟ بياكل، بيجرى، .. ؟			
• **النمو الحركى الاجمالى**			
• هل ابنك بيجرى كويس إلى حد كبير، ويقف لوحده من غير ما يتكعبل أو يوقع؟			
• ٢. أثناء الوقوف, هل يرمي طفلك كرة مرفوعة عن طريق رفع ذراعه إلى ارتفاع الكتف ورمي الكرة للأمام؟			
• هل ابنك بيعرف يشوط الكورة لقدام بدون ما يبقى ساند على حاجة؟			
• هل ابنك بيعرف ينط برجليه الاتنين ويبعد عن الأرض؟			
• هل ابنك ممكن يطلع السلم وهوا مستخدم رجل وحدة على كل سلمة؟			
• هل ابنك ممكن يقف على رجل وحدة لمدة ثانية بدون ما يمسك في حاجة؟			
**النمو الحركى الدقيق**			
• هل يحاول طفلك قص الورق بمقص آمن للأطفال؟			

• لو وريت ابنك ازاى يرسم خط من أول الورقة لآخرها، بقلم شمع أو حبر أو رصاص، هل ممكن يقلدك ويرسم خط مستقيم بصورة "عمودية"؟			
• هل ممكن ابنك يدخل حبل أو رباط شوز جوه خرز كبير أو مكرونة؟			
• لو وريت ابنك ازاى يرسم خط من أول الورقة لآخرها، بقلم شمع أو حبر أو رصاص، هل ممكن يقلدك ويرسم خط مستقيم بصورة "أفقية"؟			
• لو رسمت دايرة قدام ابنك، هل ممكن يقلدك ويرسم دايرة لوحده؟			
• هل ابنك ممكن يقلب صفحات الكتاب صفحة بصفحة؟			

حل المشكلات

• وابنك باصص لنفسه في المراية، اسأله: فين .... "اسم ابنك"، هل بيشاور على صورته في المراية؟			
• لو ابنك عايز يجيب حاجة ومش طايلها، هل بيروح يجيب كرسى أو بوكس عشان يقف عليه، زى يجيب لعبة من على الترابيزة، أو يساعدك في المطبخ؟			
• لو ابنك شافك وانت بترص ٤ مكعبات أو عربيات جنب بعض صف واحد، هل ممكن يقلدك؟			
• لو شاورت على الرسمة وسألت ابنك ايه ده؟ هل ممكن يقول كلمة يقصد بيها شخص أو حاجة؟ زى رجل الثلج، ولد، راجل، بنت، بابا، سجل رد ابنك.			
• لو قلت لابنك قول "سبعة – تلاتة" أو "تمانية – اتنين" هل ابنك بيكرر وراك الرقمين بنفس الترتيب؟			
• لو ابنك رسم رسمة بسيطة وسألته ايه ده؟ هل بيقولك رسم ايه؟			

الشخصية الاجتماعية

• لو سالت طفلك, " هل أنت ولد أو بنت ?"هل يجيب طفلك بشكل صحيح?			
• هل ابنك بيأكل نفسه بالمعلقة بدون ما يوقع كتير؟			
• هل ابنك بيزق عربية أو لعبة بعجلات بين العفش وبيرجع بيها لورا؟			
• هل ابنك ممكن يلبس الجاكيت أو التيشرت لوحده؟			

- وانت بتلبس ابنك النبطلون، هل ممكن يشده لفوق لحد وسطه؟
- لما ابنك يبقى باصص لنفسه في المراية وسألته مين في المراية؟ هل بيقولك أنا أو يقول اسمه؟

اسئلة عامة نعم لا

- هل تعتقد ان ابنك بيسمع كويس ؟ لو الإجابة لأ وضح
- ....
- هل ابنك بيتكلم زي باقي الأطفال اللي في سنه؟ لو الإجابة لأ وضح؟
  -
- هل تقدر تفهم معظم كلام ابنك؟ لو الإجابة لأ وضح؟
  -
- هل ابنك بيمشي و يجري و يتسلق زي باقي الاطفال اللي في سنه؟ لو الإجابة لأ وضح؟
  -
- هل فيه تاريخ في العيلة سواء من الأب أو الأم للصم، أو مشاكل في السمع؟ لو الإجابة نعم وضح؟
  -
- هل عندك قلق عن نظر ابنك؟ لو الإجابة نعم وضح؟
  -
- هل ابنك عنده أى مشاكل صحية في آخر كام شهر؟ لو الإجابة نعم وضح؟
  -
- هل عندك أى تساؤلات عن سلوكيات ابنك؟ لو الإجابة نعم وضح؟
  -
- هل فيه أى حاجة ناحية ابنك تقلقك؟ لو الإجابة نعم وضح؟
- ....

٣٦ شهر

السؤال	نعم	احيانا	ليس بعد
**مهارات الاتصال**			
• ظهر لطفلك كيف يتحرك سوستة على معطف لأعلى ولأسفل ، وقل ، " انظر ، هذا يرتفع لأعلى ولأسفل."ضع سوستة في المنتصف واطلب من طفلك تحريك السحاب لأسفل. أعد السحاب إلى المنتصف واطلب من طفلك تحريك سوستة لأعلى. افعل ذلك عدة مرات	☐	☐	☐
• عندما تسأل, "ما هو اسمك؟"هل يقول طفلك اسمه الأول أو لقبه؟	☐	☐	☐
• لو طلبت من ابنك يشاور على أجزاء جسمه، زى الأنف، العينين، الشعر، الودان، هل بيشاور على ٧ أجزاء على الأقل؟ "ممكن يشاور على نفسه، أو على باباه، أو على لعبة" "لو شاور على ٣ بس اختار أحيانا".	☐	☐	☐
• هل ابنك بيكون جمل من ٣ أو ٤ كلمات؟ ادينى مثال؟	☐	☐	☐
• بدون ما تساعد ابنك، اسأله: حط الكتاب على الترابيزة، حط الشوز تحت الكرسى، هل ممكن ينفذ الأوامر دى بطريقة صحيحة؟	☐	☐	☐
• لو وريت ابنك صورة في كتاب وسألته: الولد بيعمل ايه؟ هل بيجاوب بالفعل الصحيح؟ بياكل، بيجرى، .. ؟	☐	☐	☐
**النمو الحركي الاجمالي**			
• ١.هل يقفز طفلك للأمام على الأقل ١٥ سنتيمتر مع وصول كلا القدمين للأرض في نفس الوقت؟	☐	☐	☐
• ٢. أثناء الوقوف, هل يرمي طفلك كرة مرفوعة عن طريق رفع ذراعه إلى ارتفاع الكتف ورمي الكرة للأمام؟	☐	☐	☐
• هل ابنك بيعرف يشوط الكورة لقدام بدون ما يبقى ساند على حاجة؟	☐	☐	☐
• هل ابنك بيعرف ينط برجليه الاتنين ويبعد عن الأرض؟	☐	☐	☐
• هل ابنك ممكن يطلع السلم وهوا مستخدم رجل وحدة على كل سلمة؟	☐	☐	☐
• هل ابنك ممكن يقف على رجل وحدة لمدة ثانية بدون ما يمسك	☐	☐	☐

في حاجة؟

النمو الحركى الدقيق

- هل يحاول طفلك قص الورق بمقص آمن للأطفال؟
- لو وريت ابنك ازاى يرسم خط من أول الورقة لآخرها، بقلم شمع أو حبر أو رصاص، هل ممكن يقلدك ويرسم خط مستقيم بصورة "عمودية"؟
- هل ممكن ابنك يدخل حبل أو رباط شوز جوه خرز كبير أو مكرونة؟
- لو وريت ابنك ازاى يرسم خط من أول الورقة لآخرها، بقلم شمع أو حبر أو رصاص، هل ممكن يقلدك ويرسم خط مستقيم بصورة "أفقية"؟
- لو رسمت دايرة قدام ابنك، هل ممكن يقلدك ويرسم دايرة لوحده؟
- ٦.عند الرسم, هل يحمل طفلك قلم رصاص, قلم تلوين, أو قلم بين أصابعها وإبهامها كما يفعل الكبار؟

حل المشكلات

- ١.أظهر لطفلك كيفية إنشاء جسر به كتل أو صناديق أو علب ، مثل المثال. هل يصنع طفلك بجعل واحدة مثل ذلك
- لو ابنك عايز يجيب حاجة ومش طايلها، هل بيروح يجيب كرسى أو بوكس عشان يقف عليه، زى يجيب لعبة من على الترابيزة، أو يساعدك في المطبخ؟
- لو ابنك شافك وانت بترص ٤ مكعبات أو عربيات جنب بعض صف واحد، هل ممكن يقلدك؟
- لو شاورت على الرسمة وسألت ابنك ايه ده؟ هل ممكن يقول كلمة يقصد بيها شخص أو حاجة؟ زى رجل الثلج، ولد، راجل، بنت، بابا، سجل رد ابنك.
- لو قلت لابنك قول "سبعة – تلاتة" أو "تمانية – اتنين" هل ابنك بيكرر وراك الرقمين بنفس الترتيب؟
- ٦.هل يكرر طفلك الأرقام الثلاثة فقط عندما تقولها بنفس الترتيب؟

الشخصية الاجتماعية

- ١.لو سالت طفلك, " هل أنت ولد أو بنت ؟"هل يجيب طفلك بشكل صحيح؟
- ٢.هل ابنك بيأكل نفسه بالمعلقة بدون ما يوقع كتير؟

- ٣.هل ابنك بيزق عربية أو لعبة بعجلات بين العفش وبيرجع بيها لورا؟
- ٤.هل ابنك ممكن يلبس الجاكيت أو التيشرت لوحده؟
- هل يستنى الدور عن طريق الانتظار
- لما ابنك يبقى باصص لنفسه في المراية وسألته مين في المراية؟ هل بيقولك أنا أو يقول اسمه؟

اسئلة عامة نعم لا

- هل تعتقد ان ابنك بيسمع كويس ؟ لو الإجابة لأ وضح
- ....
- هل ابنك بيتكلم زي باقي الأطفال اللي في سنه؟ لو الإجابة لأ وضح؟
- هل تقدر تفهم معظم كلام ابنك؟ لو الإجابة لأ وضح؟
- هل ابنك بيمشي و يجري و يتسلق زي باقي الاطفال اللي في سنه؟ لو الإجابة لأ وضح؟
- هل فيه تاريخ في العيلة سواء من الأب أو الأم للصم، أو مشاكل في السمع؟ لو الإجابة نعم وضح؟
- هل عندك قلق عن نظر ابنك؟ لو الإجابة نعم وضح؟
- هل ابنك عنده أى مشاكل صحية في آخر كام شهر؟ لو الإجابة نعم وضح؟
- هل عندك أى تساؤلات عن سلوكيات ابنك؟ لو الإجابة نعم وضح؟
- هل فيه أى حاجة ناحية ابنك تقلقك؟ لو الإجابة نعم وضح؟
- ....

٤٢ شهر

السؤال	نعم	احيانا	ليس بعد
**مهارات الاتصال**			
• ظهر لطفلك كيف يتحرك سوستة على معطف لأعلى ولأسفل ، وقل ، " انظر ، هذا يرتفع لأعلى ولأسفل."ضع سوستة في المنتصف واطلب من طفلك تحريك السحاب لأسفل. أعد السحاب إلى المنتصف واطلب من طفلك تحريك سوستة لأعلى. افعل ذلك عدة مرات			
• عندما تسأل, "ما هو اسمك؟"هل يقول طفلك اسمه الأول أو لقبه؟			
• من خلال الإشارة أو تكرار الاتجاهات, هل يتبع طفلك ثلاثة اتجاهات لا علاقة لها ببعضها البعض؟			
• ٤. هل يستخدم طفلك كل الكلمات ليصنع الجملة كاملة؟			
• بدون ما تساعد ابنك، اسأله: حط الكتاب على الترابيزة، حط الشوز تحت الكرسى، هل ممكن ينفذ الأوامر دى بطريقة صحيحة؟			
• لو وريت ابنك صورة في كتاب وسألته: الولد بيعمل ايه؟ هل بيجاوب بالفعل الصحيح؟ بياكل، بيجرى، .. ؟			
**النمو الحركي الاجمالي**			
• ١.هل يقفز طفلك للأمام على الأقل ١٥ سنتيمتر مع وصول كلا القدمين للأرض في نفس الوقت؟			
• ٢. أثناء الوقوف, هل يرمي طفلك كرة مرفوعة عن طريق رفع ذراعه إلى ارتفاع الكتف ورمي الكرة للأمام؟			
• هل يتسلق طفلك درجات سلم الزحليقة وينزلق لأسفل دون مساعدة؟			
• هل يمسك طفلك كرة كبيرة بكلتا يديه؟			
• هل ابنك ممكن يطلع السلم وهوا مستخدم رجل وحدة على كل سلمة؟			
• هل ابنك ممكن يقف على رجل وحدة لمدة ثانية بدون ما يمسك في حاجة؟			
**النمو الحركى الدقيق**			
• هل يحاول طفلك قص الورق بمقص آمن للأطفال؟			

- استخدام الشكل خطين متقاطعين, هل ينسخها طفلك على قطعة كبيرة من الورق باستخدام قلم رصاص, قلم تلوين, أو قلم, بدون تتبع؟
- هل وضع طفلك معا خمسة-إلى سبع قطع اللغز المتشابكة؟
- لو وريت ابنك ازاى يرسم خط من أول الورقة لآخرها، بقلم شمع أو حبر أو رصاص، هل ممكن يقلدك ويرسم خط مستقيم بصورة "أفقية"؟
- لو رسمت دايرة قدام ابنك، هل ممكن يقلدك ويرسم دايرة لوحده؟
- ٦.عند الرسم, هل يحمل طفلك قلم رصاص, قلم تلوين, أو قلم بين أصابعها وإبهامها كما يفعل الكبار؟

حل المشكلات

- ١.أظهر لطفلك كيفية إنشاء جسر به كتل أو صناديق أو علب ، مثل المثال. هل يصنع طفلك بجعل واحدة مثل ذلك
- عندما سؤاله, "أي دائرة هي الأصغر؟"هل يشير طفلك إلى أصغر دائرة؟
- هل يرتدي طفلك ملابسه و "يلعب دور" ، متظاهرا بأنه شخص أو شيء آخر؟
- لو شاورت على الرسمة وسألت ابنك ايه ده؟ هل ممكن يقول كلمة يقصد بيها شخص أو حاجة؟ زى رجل الثلج، ولد، راجل، بنت، بابا، سجل رد ابنك.
- لو قلت لابنك قول "سبعة – تلاتة" أو "تمانية – اتنين" هل ابنك بيكرر وراك الرقمين بنفس الترتيب؟
- ٦.هل يكرر طفلك الأرقام الثلاثة فقط عندما تقولها بنفس الترتيب؟

الشخصية الاجتماعية

- ١.لو سالت طفلك, " هل أنت ولد أو بنت ؟"هل يجيب طفلك بشكل صحيح؟
- ٢. هل يغسل طفلك يديه باستخدام الماء والصابون ويجف بمنشفة دون مساعدة؟
- ٣. هل يخدم طفلك نفسه ، ويأخذ الطعام من حاوية إلى أخرى باستخدام الأواني؟
- ٤.هل ابنك ممكن يلبس الجاكيت أو التيشرت لوحده؟
- ٥. هل يستنى الدور عن طريق الانتظار

- لما ابنك يبقى باصص لنفسه في المراية وسألته مين في المراية؟ هل بيقولك أنا أو يقول اسمه؟ ☐ ☐ ☐

اسئلة عامة

	نعم	لا
٢. هل تعتقد ان ابنك بيسمع كويس ؟ لو الإجابة لأ وضح	☐	☐
٣. هل ابنك بيتكلم زي باقي الأطفال اللي في سنه؟ لو الإجابة لأ وضح؟	☐	☐
٤. هل تقدر تفهم معظم كلام ابنك؟ لو الإجابة لأ وضح؟	☐	☐
٥. هل ابنك بيمشي و يجري و يتسلق زي باقي الاطفال اللي في سنه؟ لو الإجابة لأ وضح؟	☐	☐
٦. هل فيه تاريخ في العيلة سواء من الأب أو الأم للصم، أو مشاكل في السمع؟ لو الإجابة نعم وضح؟	☐	☐
٧. هل عندك قلق عن نظر ابنك؟ لو الإجابة نعم وضح؟	☐	☐
٨. هل ابنك عنده أى مشاكل صحية في آخر كام شهر؟ لو الإجابة نعم وضح؟	☐	☐
٩. هل عندك أى تساؤلات عن سلوكيات ابنك؟ لو الإجابة نعم وضح؟	☐	☐
١٠. هل فيه أى حاجة ناحية ابنك تقلقك؟ لو الإجابة نعم وضح؟	☐	☐

48 شهر

السؤال   نعم   احيانا   ليس بعد

<u>مهارات الاتصال</u>

- هل يقوم طفلك بتسمية ثلاثة عناصر على الأقل من فئة مشتركة؟ على سبيل المثال ، إذا قلت لطفلك ، "أخبرني ببعض الأشياء التي يمكنك تناوله
- هل يجيب طفلك على الأسئلة التالية؟ ماذا تفعل عندما تكون جائعا و متعب
- من خلال الإشارة أو تكرار الاتجاهات, هل يتبع طفلك ثلاثة اتجاهات لا علاقة لها ببعضها البعض؟
- هل يستخدم طفلك كل الكلمات ليصنع الجملة كاملة؟
- هل يخبرك طفلك بشيئين على الأقل عن الأشياء الشائعة؟ على سبيل المثال ، إذا قلت لطفلك ، "أخبرني عن كرتك،"
- لو وريت ابنك صورة في كتاب وسألته: الولد بيعمل ايه؟ هل بيجاوب بالفعل الصحيح؟ بياكل، بيجرى، .. ؟
- <u>النمو الحركى الاجمالى</u>
- ١.هل يقفز طفلك للأمام على الأقل ٢٠ سنتيمتر مع وصول كلا القدمين للأرض في نفس الوقت؟
- ٢. أثناء الوقوف, هل يرمي طفلك كرة مرفوعة عن طريق رفع ذراعه إلى ارتفاع الكتف ورمي الكرة للأمام؟
- هل يتسلق طفلك درجات سلم الزحليقة وينزلق لأسفل دون مساعدة؟
- هل يمسك طفلك كرة كبيرة بكلتا يديه؟
- هل يقفز طفلك لأعلى ولأسفل على القدم اليمنى أو اليسرى مرة واحدة على الأقل دون أن يفقد توازنه أو يسقط؟
- هل ابنك ممكن يقف على رجل وحدة لمدة ٥ ثوانى بدون ما يمسك في حاجة؟

<u>النمو الحركى الدقيق</u>

- هل يحاول طفلك قص الورق بمقص آمن للأطفال؟
- استخدام الشكل خطين متقاطعين, هل ينسخها طفلك على قطعة كبيرة من الورق باستخدام قلم رصاص, قلم تلوين, أو قلم, بدون تتبع؟

- هل وضع طفلك معا خمسة-إلى سبع قطع اللغز المتشابكة؟
- هل يقوم طفلك بفك زر واحد أو أكثر؟
- هل يرسم طفلك صورا لأشخاص لديهم ثلاث من الميزات التالية على الأقل: رأس, عيون, أنف, فم, العنق, شعر, جذع, أسلحة, أيادي, أرجل, أو القدمين؟
- هل لون طفلك في الغالب داخل خطوط في كتاب التلوين أو داخل خطوط دائرة ٢ بوصة التي ترسمها؟

حل المشكلات

- عندما تظهر الكائنات وسأل" " ما هو لون هذا؟"هل اسم طفلك خمسة ألوان مختلفة, مثل الأحمر, أزرق, الأصفر, البرتقالي, أسود, أبيض, أو الوردي؟
- عندما سؤاله, "أي دائرة هي الأصغر؟"هل يشير طفلك إلى أصغر دائرة؟
- هل يرتدي طفلك ملابسه و "يلعب دور" ، متظاهرا بأنه شخص أو شيء آخر؟
- إذا وضعت خمسة أشياء أمام طفلك, هل يمكنه عدها بقول," واحد, اثنان, ثلاثة, أربعة, خمسة, " بالترتيب؟.
- بدون تقديم المساعدة من خلال الإشارة, هل يتبع طفلك ثلاثة اتجاهات مختلفة باستخدام الكلمات" تحت, "" بين, "و"وسط"؟ على سبيل المثال ، اطلب من طفلك وضع الحذاء "تحت الأريكة."
- هل يكرر طفلك الأرقام الثلاثة فقط عندما تقولها بنفس الترتيب؟

الشخصية الاجتماعية

- هل يخبرك طفلك بأربعة على الأقل مما يلي؟ يرجى وضع علامة على العناصر التي يعرفها طفلك.

أ. الاسم الأول د. اسم العائلة

ب. العمر ه. صبي أو فتاة

ج. المدينة التي تعيش فيها

د. رقم الهاتف

- هل يغسل طفلك يديه باستخدام الماء والصابون ويجف بمنشفة دون مساعدة؟

- هل يخدم طفلك نفسه ، ويأخذ الطعام من حاوية إلى أخرى باستخدام الأواني؟
- هل ابنك ممكن يلبس الجاكيت أو التيشرت لوحده؟
- هل طفلك يغسل اسنانه عن طريق وضع معجون الأسنان على فرشاة الأسنان وتنظيف جميع أسنانها دون مساعدة؟
- هل يخبرك طفلك بأسماء اثنين أو أكثر من زملائه في اللعب, لا يشمل الإخوة والأخوات؟

<u>اسئلة عامة</u> نعم لا

- هل تعتقد ان ابنك بيسمع كويس ؟ لو الإجابة لأ وضح
- ....
- هل ابنك بيتكلم زي باقي الأطفال اللي في سنه؟ لو الإجابة لأ وضح؟
- هل تقدر تفهم معظم كلام ابنك؟ لو الإجابة لأ وضح؟
- هل ابنك بيمشي و يجري و يتسلق زي باقي الاطفال اللي في سنه؟ لو الإجابة لأ وضح؟
- هل فيه تاريخ في العيلة سواء من الأب أو الأم للصم، أو مشاكل في السمع؟ لو الإجابة نعم وضح؟
- هل عندك قلق عن نظر ابنك؟ لو الإجابة نعم وضح؟
- هل ابنك عنده أى مشاكل صحية في آخر كام شهر؟ لو الإجابة نعم وضح؟
- هل عندك أى تساؤلات عن سلوكيات ابنك؟ لو الإجابة نعم وضح؟
- هل فيه أى حاجة ناحية ابنك تقلقك؟ لو الإجابة نعم وضح؟
- ....

٥٤ شهر

السؤال | نعم | احيانا | ليس بعد

مهارات الاتصال

- **هل يستخدم طفلك أربع-والجمل خمس كلمات؟ على سبيل المثال ، هل يقول طفلك ، "أريد السيارة"؟**
- **هل يستخدم طفلك نهايات الكلمات بطريقة صحيحة**
- **من خلال الإشارة أو تكرار الاتجاهات, هل يتبع طفلك ثلاثة اتجاهات لا علاقة لها ببعضها البعض؟**
- **هل يستخدم طفلك كل الكلمات ليصنع الجملة كاملة؟**
- **هل يخبرك طفلك بشيئين على الأقل عن الأشياء الشائعة؟ على سبيل المثال ، إذا قلت لطفلك ، "أخبرني عن كرتك،"**
- **لو وريت ابنك صورة في كتاب وسألته: الولد بيعمل ايه؟ هل بيجاوب بالفعل الصحيح؟ بياكل، بيجرى، .. ؟**
- **النمو الحركي الاجمالي**
- **هل يقفز طفلك للأمام على الأقل ٢٠ سنتيمتر مع وصول كلا القدمين للأرض في نفس الوقت؟**
- **أثناء الوقوف, هل يرمي طفلك كرة مرفوعة عن طريق رفع ذراعه إلى ارتفاع الكتف ورمي الكرة للأمام؟**
- **هل يمشي طفلك على أطراف أصابعه لمسافة ١٥ قدما**
- **هل يمسك طفلك كرة كبيرة بكلتا يديه؟**
- **هل يقفز طفلك لأعلى ولأسفل على القدم اليمنى أو اليسرى مرة واحدة على الأقل دون أن يفقد توازنه أو يسقط؟**
- **هل ابنك ممكن يقف على رجل وحدة لمدة ٥ ثوانى بدون ما يمسك في حاجة؟**

النمو الحركي الدقيق

- **هل يحاول طفلك قص الورق بمقص آمن للأطفال؟**
- **هل ينسخ طفلك ثلاثة أشكال على الأقل على قطعة كبيرة من الورق باستخدام قلم رصاص, قلم تلوين, أو قلم, بدون تتبع؟**
- **اطلب من طفلك أن يتتبع على الخط المستقيم بقلم رصاص. هل تتبع طفلك على الخط دون الخروج عن الخط أكثر من مرتين؟**
- **هل يقوم طفلك بفك زر واحد أو أكثر؟**

- اطلب من طفلك رسم صورة لشخص على ورقة بيضاء. قد تسأل طفلك، " ارسم صورة لفتاة أو صبي."إذا رسم طفلك شخصا برأسه وجسمه وذراعيه وساقيه ، ضع علامة" نعم."إذا رسم طفلك شخصا بثلاثة أجزاء فقط (الرأس أو الجسم أو الذراعين أو الساقين) ، ضع علامة "في بعض الأحيان."إذا رسم طفلك شخصا بجزئين أو أقل (الرأس أو الجسم أو الذراعين أو الساقين) ، ضع علامة "ليس بعد."

- هل لون طفلك في الغالب داخل خطوط في كتاب التلوين أو داخل خطوط دائرة ٢ بوصة التي ترسمها؟

حل المشكلات

- عندما تظهر الكائنات وسأل" " ما هو لون هذا؟"هل اسم طفلك خمسة ألوان مختلفة, مثل الأحمر, أزرق, الأصفر, البرتقالي, أسود, أبيض, أو الوردي؟

- عندما سؤاله, "أي دائرة هي الأصغر؟"هل يشير طفلك إلى أصغر دائرة؟

- هل يرتدي طفلك ملابسه و "يلعب دور" ، متظاهرا بأنه شخص أو شيء آخر؟

- إذا وضعت خمسة أشياء أمام طفلك, هل يمكنه عدها بقول," واحد, اثنان, ثلاثة, أربعة, خمسة, " بالترتيب؟.

- هل يصل عدد طفلك إلى ١٥ دون ارتكاب أخطاء؟ إذا كان الأمر كذلك ، ضع علامة " نعم."إذا كان طفلك يحسب إلى ١٢ دون ارتكاب أخطاء ، ضع علامة" في بعض الأحيان."

- هل يعرف طفلك أسماء الأرقام؟ (ضع علامة " نعم " إذا حدد الأرقام الثلاثة أدناه. ضع علامة "أحيانا" إذا حدد رقمين.)؟

الشخصية الاجتماعية

- هل يخبرك طفلك بأربعة على الأقل مما يلي؟ يرجى وضع علامة على العناصر التي يعرفها طفلك.

أ. الاسم الأول د. اسم العائلة

ب. العمر ه. صبي أو فتاة

ج. المدينة التي تعيش فيها
د. رقم الهاتف

- هل يغسل طفلك يديه باستخدام الماء والصابون ويجف بمنشفة

دون مساعدة؟

- هل يخدم طفلك نفسه ، ويأخذ الطعام من حاوية إلى أخرى باستخدام الأواني؟
- هل يرتدي طفلك ملابسه ويخلع ملابسه بنفسه, بما في ذلك الأزرار متوسطة الحجم والسحابات الأمامية؟
- هل طفلك يغسل اسنانه عن طريق وضع معجون الأسنان على فرشاة الأسنان وتنظيف جميع أسنانها دون مساعدة؟
- هل يخبرك طفلك بأسماء اثنين أو أكثر من زملائه في اللعب, لا يشمل الإخوة والأخوات؟

اسئلة عامة

نعم لا

- هل تعتقد ان ابنك بيسمع كويس ؟ لو الإجابة لأ وضح
- ....
- هل ابنك بيتكلم زي باقي الأطفال اللي في سنه؟ لو الإجابة لأ وضح؟
- هل تقدر تفهم معظم كلام ابنك؟ لو الإجابة لأ وضح؟
- هل ابنك بيمشي و يجري و يتسلق زي باقي الاطفال اللي في سنه؟ لو الإجابة لأ وضح؟
- هل فيه تاريخ في العيلة سواء من الأب أو الأم للصم، أو مشاكل في السمع؟ لو الإجابة نعم وضح؟
- هل عندك قلق عن نظر ابنك؟ لو الإجابة نعم وضح؟
- هل ابنك عنده أى مشاكل صحية في آخر كام شهر؟ لو الإجابة نعم وضح؟
- هل عندك أى تساؤلات عن سلوكيات ابنك؟ لو الإجابة نعم وضح؟
- هل فيه أى حاجة ناحية ابنك تقلقك؟ لو الإجابة نعم وضح؟
- ....
